# Naomi: a new modelling tool for estimating HIV epidemic indicators at the district level in sub‐Saharan Africa

**DOI:** 10.1002/jia2.25788

**Published:** 2021-09-21

**Authors:** Jeffrey W. Eaton, Laura Dwyer‐Lindgren, Steve Gutreuter, Megan O'Driscoll, Oliver Stevens, Sumali Bajaj, Rob Ashton, Alexandra Hill, Emma Russell, Rachel Esra, Nicolas Dolan, Yusuf O. Anifowoshe, Mark Woodbridge, Ian Fellows, Robert Glaubius, Emily Haeuser, Taylor Okonek, John Stover, Matthew L. Thomas, Jon Wakefield, Timothy M. Wolock, Jonathan Berry, Tomasz Sabala, Nathan Heard, Stephen Delgado, Andreas Jahn, Thokozani Kalua, Tiwonge Chimpandule, Andrew Auld, Evelyn Kim, Danielle Payne, Leigh F. Johnson, Richard G. FitzJohn, Ian Wanyeki, Mary I. Mahy, Ray W. Shiraishi

**Affiliations:** ^1^ MRC Centre for Global Infectious Disease Analysis School of Public Health Imperial College London London UK; ^2^ Instituted for Health Metrics and Evaluation University of Washington Seattle Washington USA; ^3^ Department of Health Metrics Sciences University of Washington Seattle Washington USA; ^4^ Division of Global HIV and Tuberculosis Centers for Disease Control and Prevention Atlanta Georgia USA; ^5^ Department of Genetics University of Cambridge Cambridge UK; ^6^ Department of Zoology University of Oxford Oxford UK; ^7^ Fellows Statistics San Diego California USA; ^8^ Avenir Health Glastonbury Connecticut USA; ^9^ Department of Biostatistics University of Washington Seattle Washington USA; ^10^ Joint Centre for Excellence in Environmental Intelligence University of Exeter and the Met Office Exeter UK; ^11^ Department of Statistics University of Washington Seattle Washington USA; ^12^ Department of Mathematics Imperial College London London UK; ^13^ Fjelltopp Padiham UK; ^14^ US Department of State Washington District of Columbia USA; ^15^ ICAP, Mailman School of Public Health Columbia University New York New York USA; ^16^ Department of HIV & AIDS Ministry of Health Lilongwe Malawi; ^17^ International Training and Education Center for Health Department of Global Health University of Washington Seattle Washington USA; ^18^ Division of Global HIV and Tuberculosis Centers for Disease Control and Prevention Lilongwe Malawi; ^19^ Centre for Infectious Disease Epidemiology and Research University of Cape Town Cape Town South Africa; ^20^ Strategic Information Department Joint United Nations Program on HIV/AIDS (UNAIDS) Geneva Switzerland

**Keywords:** Bayesian statistics, HIV estimates, joint modelling, routine data, small‐area estimation

## Abstract

**Introduction:**

HIV planning requires granular estimates for the number of people living with HIV (PLHIV), antiretroviral treatment (ART) coverage and unmet need, and new HIV infections by district, or equivalent subnational administrative level. We developed a Bayesian small‐area estimation model, called Naomi, to estimate these quantities stratified by subnational administrative units, sex, and five‐year age groups.

**Methods:**

Small‐area regressions for HIV prevalence, ART coverage and HIV incidence were jointly calibrated using subnational household survey data on all three indicators, routine antenatal service delivery data on HIV prevalence and ART coverage among pregnant women, and service delivery data on the number of PLHIV receiving ART. Incidence was modelled by district‐level HIV prevalence and ART coverage. Model outputs of counts and rates for each indicator were aggregated to multiple geographic and demographic stratifications of interest. The model was estimated in an empirical Bayes framework, furnishing probabilistic uncertainty ranges for all output indicators. Example results were presented using data from Malawi during 2016–2018.

**Results:**

Adult HIV prevalence in September 2018 ranged from 3.2% to 17.1% across Malawi's districts and was higher in southern districts and in metropolitan areas. ART coverage was more homogenous, ranging from 75% to 82%. The largest number of PLHIV was among ages 35 to 39 for both women and men, while the most untreated PLHIV were among ages 25 to 29 for women and 30 to 34 for men. Relative uncertainty was larger for the untreated PLHIV than the number on ART or total PLHIV. Among clients receiving ART at facilities in Lilongwe city, an estimated 71% (95% CI, 61% to 79%) resided in Lilongwe city, 20% (14% to 27%) in Lilongwe district outside the metropolis, and 9% (6% to 12%) in neighbouring Dowa district. Thirty‐eight percent (26% to 50%) of Lilongwe rural residents and 39% (27% to 50%) of Dowa residents received treatment at facilities in Lilongwe city.

**Conclusions:**

The Naomi model synthesizes multiple subnational data sources to furnish estimates of key indicators for HIV programme planning, resource allocation, and target setting. Further model development to meet evolving HIV policy priorities and programme need should be accompanied by continued strengthening and understanding of routine health system data.

## 1 INTRODUCTION

The global community has established ambitious goals for expanding coverage of antiretroviral treatment (ART) and reducing new HIV infections to end AIDS as a public health threat by 2030 [1]. To reach these goals, HIV planning and resource allocation are increasingly undertaken at the district level where health services are typically administered, particularly in countries in sub‐Saharan Africa with the largest HIV epidemics. Establishing meaningful targets requires accurate and current estimates for the burden of HIV, new infections, and existing ART coverage in priority locations, sexes and age groups.

HIV programmes in sub‐Saharan Africa annually produce national estimates of HIV epidemic trends using the UNAIDS‐supported Spectrum and Estimation and Projection Package (EPP) models [2]. Spectrum is an integrated demographic and HIV epidemic projection model that estimates rates of HIV infection, disease progression, and mortality by sex and age since the start of the HIV epidemic, accounting for the impact of ART and prevention of mother‐to‐child transmission programmes [3, 4]. EPP statistically estimates HIV transmission rates throughout the epidemic to infer HIV incidence trends from HIV prevalence data [5].

The main challenge for robust subnational estimates is sparse data at the district level. HIV prevalence estimates from household surveys are reasonably precise at the national level, but the sample size within each district is often very small. Spatial smoothing and geostatistical models have been applied effectively to estimate subnational HIV prevalence and the number of people living with HIV (PLHIV) [6, 7, 8, 9, 10, 11, 12]. HIV prevalence among pregnant women from antenatal clinic (ANC) sentinel surveillance or routine HIV testing of ANC clients has consistently been found to be an informative predictor of spatial variation in population‐wide HIV prevalence [9, 10, 13, 14, 15, 16].

Quantifying ART coverage and unmet need in subnational areas has received less attention. Recent household surveys have included biomarker tests for antiretroviral use, which may inform spatial patterns of treatment coverage [17, 18]. National treatment coverage is conventionally estimated by comparing the number currently receiving ART from national health information systems with independently modelled estimates for the number of PLHIV [19]. This approach may lead to inaccurate estimates at finer geographic levels due to imprecision of the PLHIV denominator or ART clients attending health facilities outside their district of residence [20, 21].

To systematically utilize district‐level HIV data about multiple outcomes of interest, we created a new statistical model called Naomi, which triangulates data on population, household surveys, ART service delivery and HIV testing of pregnant women at ANCs. The model produces estimates and probabilistic uncertainty ranges for HIV prevalence, ART coverage, and the HIV incidence rate stratified by sex, age group and areas of health administration. Model application was demonstrated using example datasets from Malawi.

## 2 METHODS

The model synthesized HIV data from national household surveys and routine service provision data in a Bayesian small‐area estimation framework. Data were stratified by the subnational units at which the health system is administered, often second administrative level or “district” level. Model inputs comprised six data sources summarized in Box 1. This section provides an overview of the model. Technical details are described in Appendix S1.

**Box 1. Summary of Naomi model inputs and model outputs. (ANC, antenatal clinic; ART, antiretroviral therapy; PLHIV, people living with HIV)**

** Model inputs**

**Area hierarchy**: List of administrative areas used for health planning (“districts”), geographic boundaries, and nesting in higher level administrative areas.

**Population**: Population estimate stratified by district, sex, and five‐year age group (0 to 4, …, 80+) over the period from the most recent household survey to short‐term one‐year ahead projection.

**Household survey**: Data on HIV prevalence, ART coverage, and recent HIV infection (if available) from most recent HIV household survey, tabulated by district, sex and five‐year age group.

**Routine ANC testing: data on routine ANC HIV testing indicators by district and year**: (i) number of ANC clients (first visit), (ii) number known positive at first ANC, (iii) number already on ART prior to first ANC, (iv) number tested for HIV, and (v) number tested HIV positive.

**ART service delivery data**: Number receiving ART at health facilities in each district at the end of each calendar quarter stratified by children (0 to 14 years), adult women (15+ years) and adult men.

**Spectrum estimates**: The following outputs are exported from the national or first administrative level Spectrum files by sex and single‐year age: population size, PLHIV, number on ART, new infections, age‐specific fertility rate, HIV prevalence among pregnant women, ART coverage among pregnant women, number PLHIV unaware of HIV‐positive status (from Shiny90).

**Model outputs**

**Indicators**: The model produces outputs for the following indicators:

- Population
- HIV prevalence/PLHIV
- ART coverage/number of residents on ART/number of untreated PLHIV
- Number and percent aware/unaware of HIV status
- Number accessing ART at health facilities in each area
- HIV incidence rate/number of annual new infections
- Annual ANC clients by HIV status and ART status at first ANC

**Stratifications**: All indicators are stratified according to

- *Geographic areas: All levels of the area hierarchy (e.g., national, province, district)*
  *Sexes*: both male, female
  *Age groups*:
- Five‐year age groups (0 to 4, …, 80+)
  0 to 14, 15 to 24, 25 to 34, 35 to 49, 50 to 64, 65+
  15 to 49, 15 to 64, 15+, 50+, all ages, <1, 1 to 4
- *Time points*: Most recent household survey (T1), current quarter (T2), short‐term projection nine months to one year (T3)

**Statistics**: Posterior summary statistics are computed for

- Mean, median, mode
- Standard deviation, 95% credible interval (quantile‐based)

### 2.1 Model components

**Figure 1:**
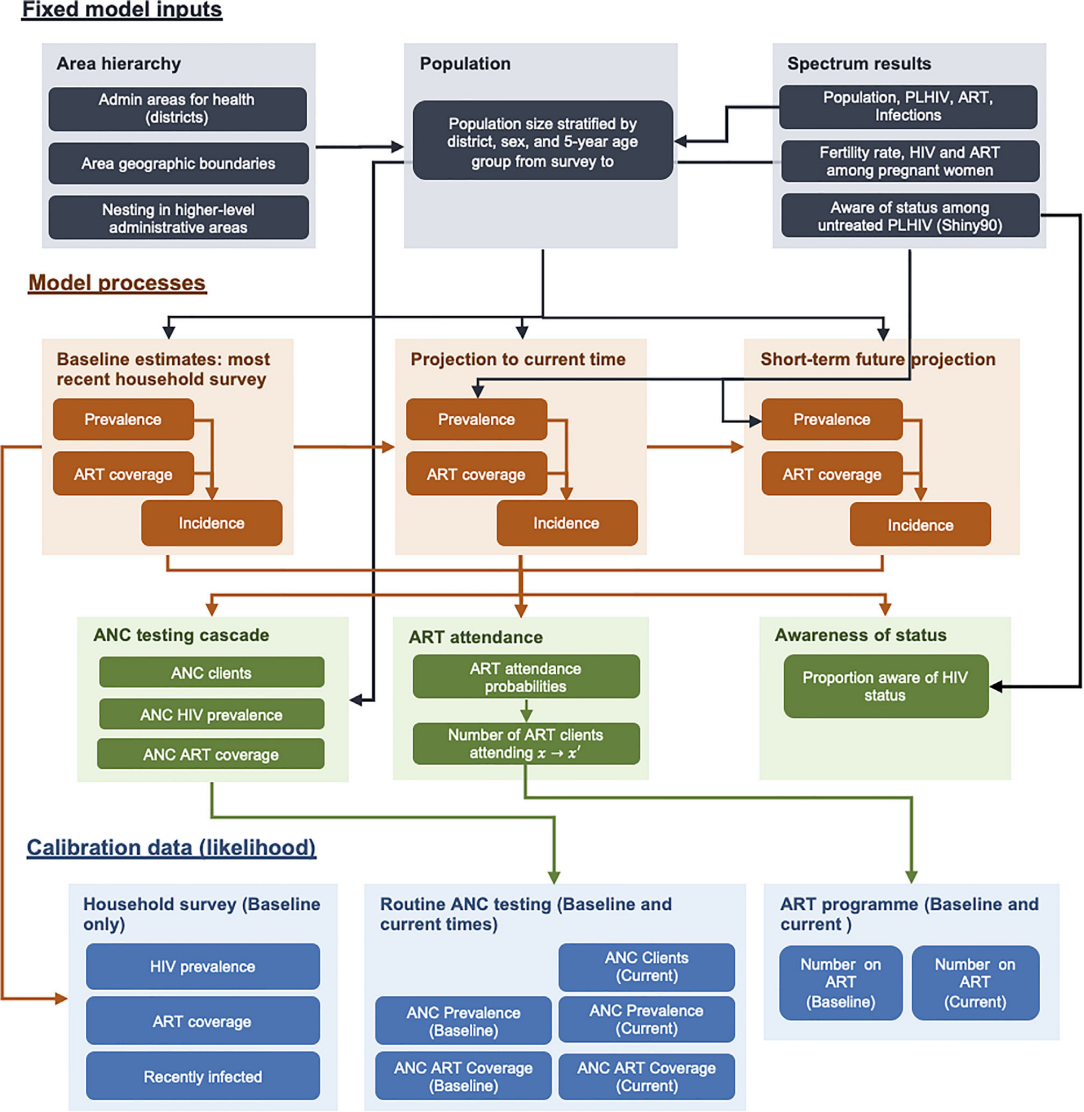
Overview of model components and processes. ANC, antenatal clinic; ART, antiretroviral treatment; PLHIV, people living with HIV.

The model involved several steps, summarized in Figure 1, to produce primary outcomes, HIV prevalence, ART coverage and HIV incidence rate by district, sex and five‐year age group at three time points. The three time points represented the time of the most recent household survey with HIV serological testing, the “current” period reflecting the most recently available HIV programme data, and a nine‐ to 12‐month future projection for short‐term programme planning.

First, small‐area estimation logistic or log‐linear regression models were specified for cross‐sectional estimates of each outcome (prevalence, ART coverage, incidence) at the time of the most recent household survey stratified by sex, five‐year age group and district. HIV prevalence and prior ART coverage among ANC clients informed spatial variation in population HIV prevalence and ART coverage. The HIV incidence rate was determined by the HIV transmission rate among untreated adults, district‐level HIV prevalence, and ART coverage. This model reflects basic HIV transmission dynamics wherein incidence risk depends on the probability of sexual contact with a partner with unsuppressed HIV infection in the local population, similar to the EPP model [5].

Second, the HIV population in each district was projected forward to the current time in a single step based on the modelled district estimates for new infections and survival rates from Spectrum estimates [3, 4].

The models for HIV prevalence and ART coverage were linked such that ART service delivery data also informed district estimates for the number of PLHIV. To account for persons receiving ART at health facilities outside their district of residence, the model estimated an odds ratio for seeking treatment in each geographically neighbouring district versus their district of residence. The predicted number of ANC clients, HIV prevalence among pregnant women and ART coverage among pregnant women were determined by the modelled sex/age‐specific district‐level HIV prevalence and ART coverage and age‐specific fertility rates and fertility rate ratios for HIV‐positive women from Spectrum.

Mother‐to‐child HIV transmissions were not explicitly modelled, but the number of children living with HIV were modelled based on the ratio of child to adult female prevalence from Spectrum results and the number of children on ART. Awareness of HIV status was modelled by applying the proportion unaware among untreated PLHIV from national Shiny90 model estimates [22] to the estimated untreated population in each district by sex and age group.

### 2.2 Model estimation

Statistical likelihood functions were specified for national household survey, routine ANC testing, and ART service delivery data. For the most recent household survey, the survey‐weighted counts for the number of HIV‐positive respondents, number on ART, and number recently infected by district, sex and five‐year age group were modelled via a binomial distribution. The likelihood for recent HIV infection data also depended on the mean duration of recent infection and false recent ratio, which are characteristics of the recent infection testing algorithm applied in a given survey [23].

ANC testing data were summarized as the total number of ANC clients, the number of clients with HIV status ascertained, the number of HIV positive (either known positive or tested positive), and the number already on ART prior to first ANC in the years of the survey and the current year. The observed number of ANC clients by district was modelled by a Poisson distribution. The observed number of HIV‐positive pregnant women and number already on ART were modelled in both years by binomial distributions.

ART service delivery data consisted of the number receiving ART at health facilities in each district for children, adult men, and adult women at the time of the survey and most recent period. The likelihood for these counts was approximated by a normal distribution, with mean and standard deviation determined by the modelled HIV prevalence, ART coverage and cross‐district ART attendance probabilities.

### 2.3 Model outputs

Model outputs consisted of counts and rates for each of the processes modelled aggregated to multiple geographic, sex and age stratifications. Box 1 summarizes output indicators. For all indicators, results were aggregated from the district level to coarser levels of the area hierarchy (e.g., provincial, national), stratified by sex and both sexes combined, and by five‐year age group and coarser priority age groups (Box 1). For the ART attendance model, outputs included the estimated number of ART clients receiving ART between each neighbouring district pair, the proportion of residents on ART who attend facilities in each neighbouring district, and the distribution of districts of residence for those receiving ART in each district. For each output, the posterior mean, median, mode, standard error and quantile‐based 95% credible intervals (CI) were reported. The posterior mean was the preferred central estimate.

#### 2.3.1 Calibration to Spectrum estimates

After fitting the model, results may optionally be adjusted such that the posterior mean of the district‐level estimates aligns to national or subnational Spectrum estimates for the number of PLHIV, on ART, new infections, and aware of HIV status. Calibrations may be applied to align by sex and coarse age groups below 15 years and 15 years and older or by five‐year age groups.

### 2.4 Implementation and inference

The model was implemented via the R package Template Model Builder (TMB), which provides analytical gradients for the posterior density via automatic differentiation and Laplace approximation of the marginal posterior [24]. The posterior distribution was approximated with an empirical Bayes strategy. One thousand samples were drawn from the joint posterior distribution, conditional on the optimized hyper‐parameters, from which the posterior mean, median, mode, standard deviation, and quantile‐based 95% CI were calculated for each output indicator.

### 2.5 Case study

We demonstrated the model using data from Malawi up to 2018. The three time points used for model estimation were March 2016 for the most recent household survey, September 2018 for the “current” time period, reflecting the most recently available ANC and ART service delivery data for this analysis, and September 2019 as a further 12‐month short‐term projection. The area hierarchy represented four levels: three regions (North/central/southern), five health zones, 28 administrative districts and 32 areas reflecting the 28 districts and four metropolitan areas (Lilongwe, Mzuzu, Blantyre, and Zomba cities). Population data were from district population projections from the 2008 household census adjusted to district population estimates from the 2018 census [25, 26]. Malawi Spectrum results were sourced from the 2019 national HIV estimates file [27].

Household survey data on HIV prevalence were used from the Malawi demographic and health survey (MDHS) 2015 to 2016 and Malawi population‐based HIV impact assessment survey (MPHIA) 2015 to 2016 [28, 29], which were conducted contemporaneously. The MPHIA furnished survey data on ART coverage and recent HIV infection. Routine data on the final HIV status of ANC clients and the number currently on ART at the end of each quarter by the 32 districts were sourced from quarterly site supervision and monitoring reports published by the Department for HIV and AIDS, Malawi Ministry of Health [30]. The total number on ART was distributed to adults (15+ years) and children (0 to 14 years), assuming 94% were adults based on Spectrum model outputs for the years 2016 to 2018.

Primary results were not calibrated to Spectrum estimates. For comparison, we also calibrated results to national Spectrum HIV estimates by sex and coarse age group (<15, 15+ years). Finally, we fitted the model without the ART attendance probabilities, assuming all ART clients attended facilities in their district of residence.

Demonstration model input datasets are available from https://github.com/mrc‐ide/naomi/tree/master/inst/extdata [31]. Demonstration datasets did not include the most current Malawi HIV programme data through 2020, and household survey inputs were constructed without reference to survey cluster geolocation datasets (see Supporting Methods for details). For the most current and complete subnational estimates produced by the Malawi HIV programme, refer to UNAIDS AIDSinfo [32]. R code reproducing the analysis is available from https://github.com/jeffeaton/naomi‐model‐paper.

## 3 RESULTS

**Figure 2:**
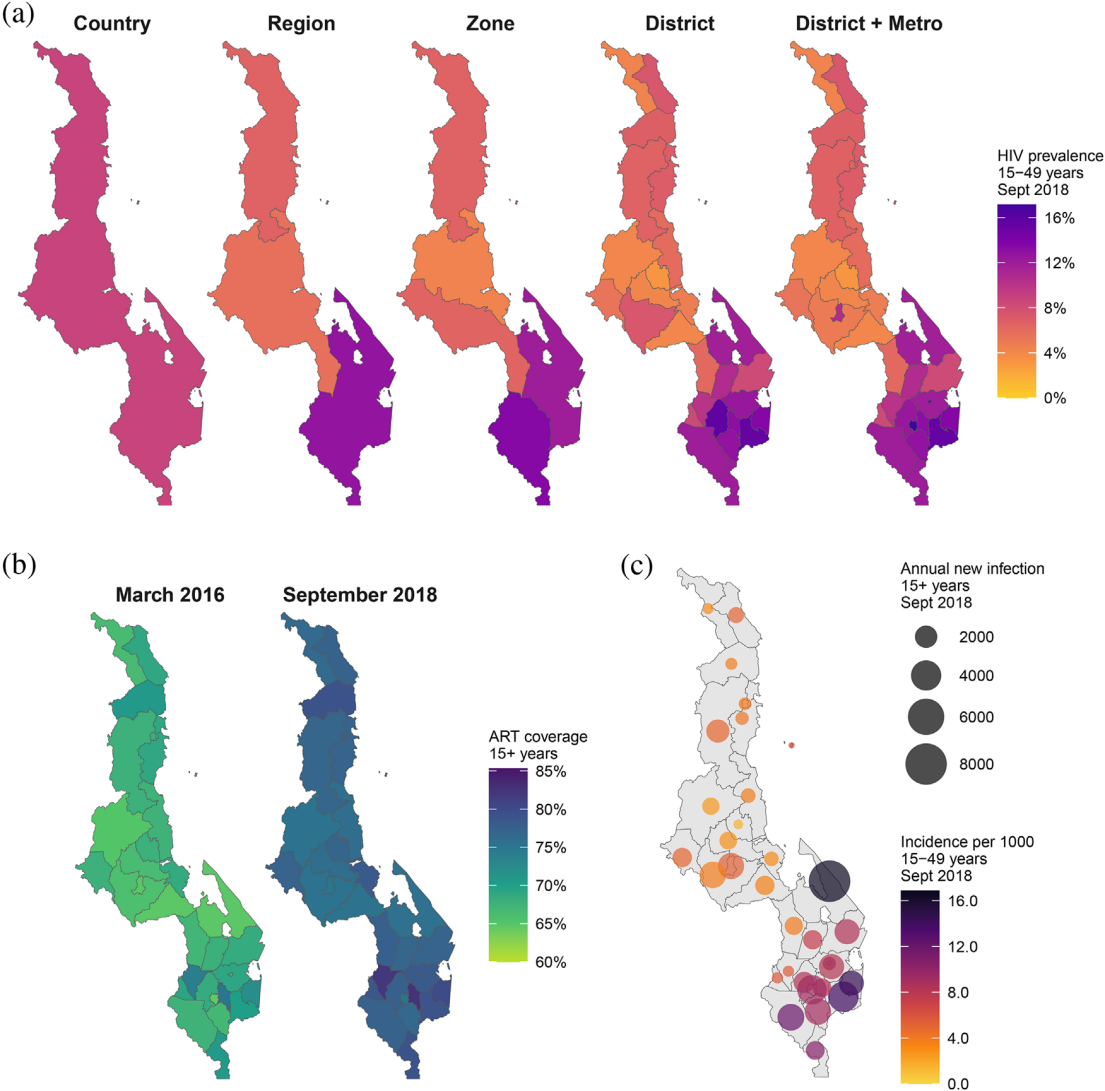
Examples of model estimates. **(a)** HIV prevalence among adults age 15 to 49 years at all levels of the area hierarchy in September 2018. **(b)** Antiretroviral treatment (ART) coverage among adults age 15 years and older by district and four metropolitan areas in March 2016 and September 2018. **(c)** HIV incidence rate among adults 15 to 49 years (colour) and annual number of new HIV infections among adults 15 years and older (size of bubble) in September 2018. Estimates reflect posterior mean. Example results did not include the most current Malawi HIV programme data, and some household survey clusters were randomly allocated to districts; refer to UNAIDS AIDSinfo for official Malawi HIV estimates [32].

Figure 2a illustrates estimates for HIV prevalence in Malawi among adults aged 15 to 49 years at all levels of the area hierarchy in September 2018. Estimated national HIV prevalence was 8.9% (95% CI, 8.4% to 9.4%). Across districts and metropolitan areas, prevalence ranged from 3.2% to 17.1% and was higher in districts in southern Malawi and higher in metropolitan areas than neighbouring districts (Lilongwe, Blantyre, and Zomba cities). The relative standard error (RSE) for district HIV prevalence estimates ranged from 4% to 16% and was lowest in Blantyre and Lilongwe cities, which were oversampled strata in the MPHIA survey.

At national level, ART coverage among adults 15 years and older increased from 67% (66% to 69%) in March 2016 to 77% (73% to 80%) in March 2018. Across districts, ART coverage was more homogenous than prevalence, ranging from 64% to 75% in 2016 and 75% to 82% in 2018 (Figure 2b). ART coverage was lowest in Blantyre city in both 2016 and 2018, but also increased by the largest amount (11% points). The estimated HIV incidence rate was higher in southern Malawi districts, with high HIV prevalence and untreated populations (Figure 2c). The RSE for the incidence rate ranged from 19% to 49%, much larger than for HIV prevalence or ART coverage. Estimated new infections were lowest in Likoma, a small island district in Lake Malawi, at 50 (20 to 95) infections per year and ranged from 230 to 8120 in mainland districts.

**Figure 3:**
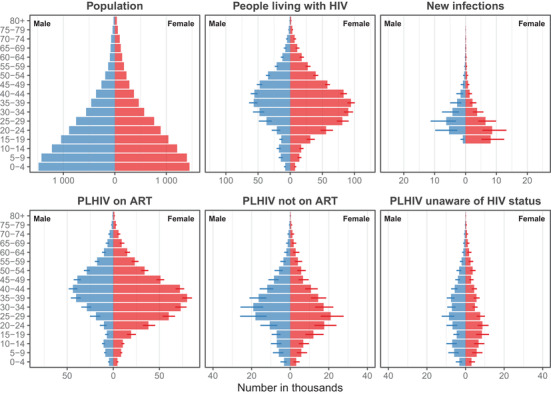
Sex and five‐year age group stratified results at national level in September 2018. Similar results are produced by region and by district. Line ranges reflect 95% credible interval ranges. Population is a fixed model input and does not has uncertainty ranges (top left). For new infections plot (top right), note that the model does not produce estimates of mother‐to‐child HIV infections, but the number of children living with HIV (top centre) are modelled based on relative levels of child to adult prevalence and paediatric antiretroviral treatment (ART) numbers. Example results did not include the most current Malawi HIV programme data, and some household survey clusters were randomly allocated to districts; refer to UNAIDS AIDSinfo for official Malawi HIV estimates [32]. PLHIV, people living with HIV.

The largest number of PLHIV was in the age group 35 to 39 years for both men and women (Figure 3). Among women, most new infections were in the age group 20 to 24 years, most untreated PLHIV were among ages 25 to 29 and the greatest number on ART were among ages 35 to 39 years. For men, the greatest number of new infections, untreated PLHIV and PLHIV on ART were in the age groups 25 to 29, 30 to 34 and 40 to 44, respectively. Relative uncertainty was much larger for the number of untreated and undiagnosed PLHIV by age (median RSE 21%) and for new infections (median RSE 29%) than for the number of PLHIV or number on ART (median RSE 10% and 9%, respectively). Similar outputs are available for each district or region.

**Figure 4:**
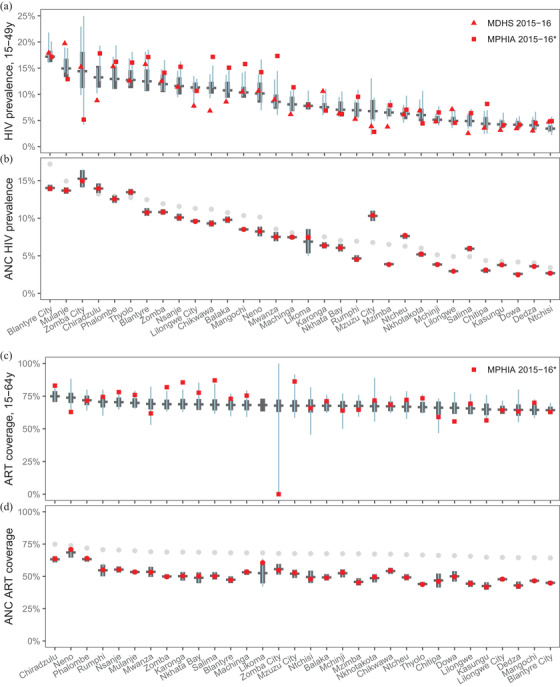
Comparison of district‐level data and model estimates for HIV prevalence and antiretroviral treatment (ART) coverage in March 2016. **(a)** HIV prevalence among adults 15 to 49 years; **(b)** HIV prevalence among antenatal clinic (ANC) clients; **(c)** ART coverage among adults 15 to 64 years; and **(d)** ART coverage prior to the first ANC visit. Thick black dash and vertical ranges show model estimates and 95% credible intervals. Narrow vertical light blue lines indicate 80% posterior predictive intervals, representing the range in which 80% probability new observations would fall. Posterior predictive ranges account for both uncertainty about true prevalence and ART coverage and sampling variability based on the sample size for each observation. For HIV prevalence results (panels a and b), districts are sequenced in decreasing order according to estimated HIV prevalence among ages 15 to 49 in March 2016. For ART coverage (panels c and d), results are sequenced in decreasing order according to estimated ART coverage ages 15 to 64 in March 2016. Red points indicate data observations from household surveys (a and c) or routine antenatal HIV testing (b and d). In (a) data points represent district HIV prevalence estimates from two surveys, with each has a different 80% posterior predictive intervals reflecting the sample size and age distribution for that survey. In ANC data plots (b and d), for comparison the light grey dots indicate posterior mean estimates for prevalence ages 15 to 49 and ART coverage ages 15 to 64 shown in panels (a) and (c), respectively. *District data for Malawi population HIV impact assessment (MPHIA) 2015 to 2016 survey are based on random allocation of survey clusters to districts within each of seven survey strata. MDHS, Malawi demographic and health survey.

Figure 4 compares district‐level estimates for HIV prevalence and ART coverage in 2016 with household survey and routine ANC testing data to which the model was calibrated. The 80% posterior predictive intervals indicate the range in which 80% of new observations would be expected to fall. For HIV prevalence, 68% (43/63) of survey observations fell within the 80% prediction interval and 87% (55/63) fell within the 95% prediction interval (Figure 4d). For ART coverage, 81% (25/31) and 97% (30/31) of observations fell within the 80% and 95% predictive ranges, respectively (Figure 4c). Estimates among ANC clients for HIV prevalence (Figure 4b) and prior ART coverage (Figure 4d) were closely calibrated to the observed values and correlated to the population prevalence and ART coverage across districts.

**Figure 5:**
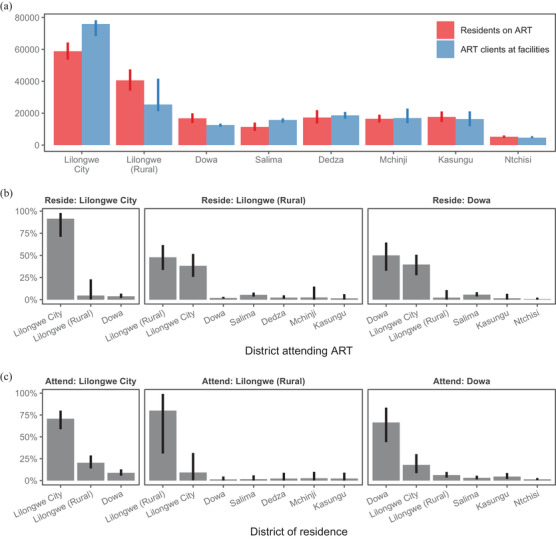
Results of antiretroviral treatment (ART) attendance model in September 2018 for three districts in central Malawi: Lilongwe city, Lilongwe district excluding the metropolitan area (rural), and Dowa district bordering Lilongwe to the north. **(a)** Estimated number of adult (age 15 years and older) residents on ART compared to number of adults receiving ART at health facilities in each district. **(b)** Percentage who receives ART at health facilities in each district by district of residence. **(c)** Distribution of district of residence for ART clients attending facilities in each district. For (b) and (c), bars are presented for all neighbouring districts. Bar heights indicate posterior mean and vertical ranges indicate 95% credible intervals. Example results did not include the most current Malawi HIV programme data, and some household survey clusters were randomly allocated to districts; refer to UNAIDS AIDSinfo for official Malawi HIV estimates [32].

Estimates for ART attendance in neighbouring districts are demonstrated in Figure 5 for Lilongwe city, Lilongwe district excluding the metropolitan area (“Lilongwe rural”), and Dowa district to the north of Lilongwe in central Malawi. In September 2018, 76,300 adults received ART at health facilities in Lilongwe city, but only 59,100 (54,100 to 64,300) resident PLHIV of Lilongwe city were on ART (Figure 5a). Conversely, health facilities in Lilongwe rural provided ART to 24,900 adults, but 40,500 (21,000 to 37,900) resident PLHIV were on ART, and Dowa facilities provided for 12,600 ART clients but 16,800 (13,800 to 20,000) residents were on ART. Thirty‐eight percent (26% to 50%) of Lilongwe rural residents and 39% (27% to 50%) of Dowa residents received treatment at health facilities in Lilongwe city (Figure 5b). Among Lilongwe city residents, 92% (76% to 98%) received treatment in the city. Among those attending facilities in Lilongwe city, an estimated 71% (61% to 79%) resided in Lilongwe city, 20% (14% to 27%) in Lilongwe rural, and 9% (6% to 12%) in Dowa (Figure 5c).

Calibration to Spectrum estimates very slightly increased the national estimate for age group 15 to 49 years HIV prevalence from 8.9% to 9.0% in September 2018; ART coverage was unchanged from 77% (Figure S1a in Additional file 1). Relative adjustments were similar across all districts (Figure S2 in Additional file 1). Calibrated prevalence decreased slightly for women and increased for men and vice versa for ART coverage. Calibrated HIV incidence was about 40% lower, with a slightly larger reduction for women than men (Figure S1a in Additional file 1). At baseline in March 2016, the calibrated estimates had slightly higher prevalence, 9.4% versus 8.8%, and lower ART coverage at 63% versus 68% uncalibrated (Figure S1b in Additional file 1). Thus, the similar prevalence and ART coverage in 2018 but lower incidence were reconciled by fewer new infections but a larger increase in ART coverage in the calibrated estimates.

Applying the model without allowing for ART attendance in neighbouring districts resulted in implausible estimates for ART coverage, PLHIV, and HIV incidence (Figure S3 in Additional file 1). When assuming that all persons on ART reside in the same district as receiving treatment, estimates for the HIV prevalence in Lilongwe city increased by 24% from 10.7% to 13.3% (Figure S3d in Additional file 1), substantially higher than the MPHIA survey estimate of 10.6% (9.4% to 12.1%). Estimated ART coverage in Lilongwe city also increased from 75% to 80% and reduced in Lilongwe rural from 75% to 65%, implying a large difference in ART coverage between Lilongwe city and Lilongwe rural, which is not consistent with ANC service delivery data.

## 4 DISCUSSION

A comprehensive HIV response requires data at the geographic level at which HIV programmes are implemented [33]. The Naomi model synthesizes multiple subnational HIV data sources to produce consistent estimates for key indicators that guide HIV resource allocation, target setting and monitoring. We have described key components of the model and presented illustrative outputs using data from Malawi. The model has been implemented as an interactive web‐based software tool [34] that provides results in under an hour, depending on the number of areas, amount of data and data consistency. The tool has been used in workshops by users from 38 sub‐Saharan African countries as part of the national HIV estimates process supported by UNAIDS.

A key innovation was to more closely integrate routinely collected HIV service delivery data into modelled district‐level HIV indicators. There is broader momentum towards relying on routine data for HIV surveillance and strategic information and strengthening health information systems [35]. Routine ANC data have been used in the EPP model to inform epidemic trends for several years [36]. We have further used these data to quantify spatial variation in HIV prevalence and, for the first time, variation in ART coverage based on the proportion of women on ART prior to the first ANC visit. Combining data about the numbers receiving ART and survey or ANC data about ART coverage leveraged routine ART service delivery data to inform estimates of the number of PLHIV in small areas, analogous to the “service multiplier” method for population size estimates [37].

Increased reliance on routinely collected service delivery data is a strength, but also has been a challenge when applying the model across countries. Model convergence and the accuracy of results are sensitive to the reliability of service delivery data inputs, especially about the number receiving ART. This should be addressed through a three‐fold strategy: (a) continued strengthening of reporting in health information systems from which data are sourced including routine data quality reviews; (b) improved data visualization and analytical tools to review input data, identify inconsistent patterns and suggest appropriate corrections; and (c) improvements to our statistical model to be more robust to improbable data and distinguish true trends from likely reporting errors. The demonstration data presented here are an example of high‐quality service delivery data curated through the successful quarterly supervision and quality improvement strategy implemented by the Department for HIV and AIDS of the Malawi Ministry of Health since 2005 [30, 38].

When modelling at small areas, allowing for some persons to obtain ART in neighbouring districts was important for reconciling district‐level data. While studies confirm this phenomenon in specific settings [39], direct data to quantify this were limited. Instead, our model relied on probabilistically triangulating data about the number on ART with data on population size, HIV prevalence and ART coverage. This required crude assumptions applying the same odds of seeking treatment in a district to all neighbouring districts. Estimates for the number seeking treatment in neighbouring districts may in some cases be a device for reconciling other data inconsistencies, for example, inaccurate district population input data. As such, these results should be interpreted cautiously. Specific data about observed or likely patterns of health system attendance are a priority to improve estimates and further realize the potential of routine health system data for HIV surveillance. Such data could include health system data on clients’ location of residence, household survey data about where PLHIV obtain care, geographic and population density data on distance to health facilities, or other population mobility data from surveys or inferred from mobile phone or transport data.

While the Naomi model used many of the same data as Spectrum and draws on Spectrum estimates for some inputs, the modelling approaches are different. Spectrum and EPP model the full history of the HIV epidemic, incorporate historical household survey and sentinel surveillance data, and leverage epidemiologic and demographic dynamics. Naomi focuses on data and estimates for the current time period and short‐term projections and incorporates data that are not currently used for calibrating Spectrum estimates, including household survey data and routine ANC data on ART coverage. When there are discrepancies between Spectrum and Naomi results, users often choose to calibrate the district‐level model results to align to Spectrum point estimates for the same indicators. These adjustments could result in misaligned estimates or targets for some locations and should be applied cautiously and with consideration. Where differences are large, we recommend reviewing the data inputs, assumptions and results of both models to identify the source of discrepancies. In our case study for Malawi, the raw Naomi results and Spectrum estimates for HIV prevalence and ART coverage were similar for September 2018, but in March 2016 the Naomi results had lower prevalence and higher ART coverage, and Spectrum estimated lower incidence at both times. The Naomi results incorporated survey data on ART coverage, which Spectrum did not, and were therefore more consistent with MPHIA survey estimates for ART coverage in 2016. Spectrum incorporated a longer data series and more mechanistic structure, which results in more stable estimates of incidence trends. Naomi incidence estimates are sensitive to inconsistent district‐level data due to sparse direct data on incidence or prevalence trends, and in many applications users exclude recent infection data to constrain implausible variation in incidence results.

Other geospatial modelling approaches for HIV have utilized spatial data on HIV risk factors and other remotely sensed data as predictive covariates and used spatially continuous approaches to modelling HIV prevalence surfaces [6, 10, 40]. For parsimony, we did not use predictive covariates in favour of focusing on modelling more HIV‐specific service delivery data sources. This decision was guided by the findings of Dwyer‐Lindgren and colleagues that inclusion of geospatial covariates only modestly improved the accuracy of spatial HIV prevalence predictions compared to a geostatistical model with only household survey and ANC sentinel surveillance [10]. Both predictive covariates and more granular spatial modelling merit further exploration, but must be balanced with the usability implications of greater data requirements and computational complexity.

Our model has several additional limitations and opportunities for further development. First, the model only produced estimates at two time points and only used data from the most recent HIV household survey. Many countries have now conducted three or more surveys with HIV testing, which other models have incorporated into spatio‐temporal modelling [10, 41]. Extending the model projection steps to earlier and finer temporal resolution is a natural way to incorporate earlier surveys and furnish annual and quarterly results, including HIV incidence trends. Second, the model assumed the age patterns of HIV prevalence, treatment coverage and incidence rates were the same across all districts. Data to inform district‐level variation in age patterns are limited. Modelling more granular age‐stratified service delivery data is an opportunity to more flexibly identify age patterns across districts, but will make estimates more sensitive to discrepancies in service delivery data inputs. Third, we assumed HIV survival was the same in all districts. District‐level data on ART retention and viral load suppression could inform variation in HIV treatment outcomes and implications for HIV deaths and PLHIV. We also did not incorporate district‐level HIV testing data into estimates of awareness of HIV status. Fourth, in cases where some data sources were not available, certain model parameters were not identifiable and consequently specified as fixed values. Fixing parameters for identifiability makes model estimates appear relatively more precise rather than more uncertain. Fifth, we did not account for uncertainty about population size by district, sex and age group, and did not explicitly model migration between districts and its impact on district prevalence and ART coverage over time. Small area population estimates are very uncertain in many countries where the most recent census was long ago or the census‐enumerated population is different from the “operational” population accessing health services in the district. Finally, the statistical calibration method did not account for uncertainty about model hyper‐parameters that determine the smoothness of model estimates, and we did not fully account for the clustered survey design. Both are ongoing areas of statistical research [42].

## 5 CONCLUSIONS

The Naomi model synthesizes data from household surveys, population and routine service delivery data at the district level to furnish estimates of key indicators with probabilistic uncertainty for HIV programme planning, resource allocation and target setting for local population areas. There are many opportunities for improving and further developing small area estimates and adapting the tools to evolving HIV policy priorities, local HIV programme needs and specific data features in given settings. Modelling of district‐level HIV service delivery data has enabled national teams to routinely review both data inputs and model outputs at subnational level, and created a feedback loop that improves the quality of both. This process should support continued strengthening of routine health system data and encourage further research about patterns of HIV care seeking to interpret these data.

## COMPETING INTERESTS

All authors declare that they have no competing interests.

## AUTHORS’ CONTRIBUTIONS

JWE, IW, MIM and RWS developed and managed the project. JWE, LD‐L, SG, MO, SB, IF, EH, TO, MLT, JW, TMW, LFJ and RWS contributed to the development of the model. MO, RA, AH, ER, ND, YLA, MW and RGF designed and developed the model software and user interface. JWE, MO, OS, RE, RG, JS, JB, TS, NH and SD prepared data inputs, reviewed data and developed specifications for data inputs. AJ, TK, TC, AA, EK and DP contributed example data and interpretation of model concepts and results. JWE wrote the first draft of the manuscript. All authors critically edited the manuscript for intellectual content and approved the final version of the manuscript for publication.

## FUNDING

This research was supported by UNAIDS, Bill & Melinda Gates Foundation (OPP1190661), National Institute of Allergy and Infectious Disease of the National Institutes of Health under award numbers R01AI136664 and R03AI125001, and the MRC Centre for Global Infectious Disease Analysis (reference MR/R015600/1), jointly funded by the UK Medical Research Council (MRC) and the UK Foreign, Commonwealth & Development Office (FCDO) under the MRC/FCDO Concordat agreement and is also part of the EDCTP2 programme supported by the European Union. TMW was supported by the Imperial College President's PhD Scholarship. RG and JS were supported by a grant from the Bill & Melinda Gates Foundation (OPP1191665). JW and TO were supported by the National Institute of Allergy and Infectious Disease of the National Institutes of Health under award number R01AI029168. This research has been supported by the President's Emergency Plan for AIDS Relief (PEPFAR) through the Centers for Disease Control and Prevention (CDC).

## DISCLAIMER

The content is solely those of the authors and does not necessarily represent the official position of the funding agencies.

## Supporting information

**Appendix S1**. Naomi: A new modelling tool for estimating HIV epidemic indicators at the district level in sub‐saharan Africa
