# Supplementary material for: Naomi: a new modelling tool for estimating HIV epidemic indicators at the district level in sub‐Saharan Africa
Source: J Int AIDS Soc. 2021 Sep 21;24(Suppl 5):e25788. doi: 10.1002/jia2.25788 (PMC8454682; doi:10.1002/jia2.25788)
Supplement: Supplementary file 1 — Appendix S1. Naomi: A new modelling tool for estimating HIV epidemic indicators at the district level in sub‐saharan Africa [file JIA2-24-e25788-s001.docx]

Naomi: A New Modelling Tool for Estimating HIV Epidemic Indicators at the District Level in Sub-Saharan Africa—Supplementary Material

Jeffrey W Eaton^§^, Laura Dwyer-Lindgren, Steve Gutreuter, Megan O’Driscoll, Oliver Stevens, Sumali Bajaj, Rob Ashton, Alexandra Hill, Emma Russell, Rachel Esra, Nicolas Dolan, Yusuf O Anifowoshe, Mark Woodbridge, Ian Fellows, Robert Glaubius, Emily Haeuser, Taylor Okonek, John Stover, Matthew L Thomas, Jon Wakefield, Timothy M Wolock, Jonathan Berry, Tomasz Sabala, Nathan Heard, Stephen Delgado, Andreas Jahn, Thokozani Kalua, Tiwonge Chimpandule, Andrew Auld, Evelyn Kim, Danielle Payne, Leigh F Johnson, Richard G FitzJohn, Ian Wanyeki, Mary Mahy, Ray W Shiraishi

^§^Corresponding author: Jeffrey W Eaton ([*jeffrey.eaton@imperial.ac.uk*](mailto:jeffrey.eaton@imperial.ac.uk))

**Table of Contents**

[Supplementary Methods 2](#_Toc77605650)

[Data inputs 2](#_Toc77605651)

[Area hierarchy 2](#_Toc77605652)

[Population 2](#_Toc77605653)

[Household survey 2](#_Toc77605654)

[ANC testing and ART service delivery data 3](#_Toc77605655)

[Spectrum estimates 3](#_Toc77605656)

[Process model 4](#_Toc77605657)

[HIV prevalence and ART coverage at T1 4](#_Toc77605658)

[HIV incidence rate 5](#_Toc77605659)

[Short-term projection from T1 to T2 and T2 to T3 6](#_Toc77605660)

[ANC testing cascade 8](#_Toc77605661)

[ART attendance 9](#_Toc77605662)

[Awareness of HIV status 10](#_Toc77605663)

[Likelihood specification 10](#_Toc77605664)

[Household survey data 10](#_Toc77605665)

[ANC testing data 11](#_Toc77605666)

[Number receiving ART 12](#_Toc77605667)

[Identifiability constraints 13](#_Toc77605668)

[Case study data 14](#_Toc77605669)

[Supplementary Figures 15](#_Toc77605670)

[Figure S1. 15](#_Toc77605671)

[Figure S2. 16](#_Toc77605672)

[Figure S3. 17](#_Toc77605673)

# Supplementary Methods

This supplementary methods section describes technical details of the data and input requirements, process model steps, and likelihood formulation outlined in Figure 1.

## Data inputs

Model inputs comprise six data sources summarised in Box 1: (1) area hierarchy, (2) population size, (3) household surveys, (4) routine ANC HIV testing data, (5) routine ART service delivery data, and (6) national or subnational Spectrum model estimates.

### Area hierarchy

The area hierarchy consists of a listing of administrative units used for health system provision (e.g. districts), area geographic boundaries, which are used to define adjacency structures for neighbouring districts, and nesting of districts within higher level statistical or administrative areas for which aggregated results are useful, for example, provinces or regions.

### Population

Required population data are population size by district, sex, and five-year age group over the period spanning the most recent household survey through a one-year-ahead projection. Data are sourced from district population projections produced by national statistical offices, household censuses, or global population data products such as the Gridded Population of the World [1] or the WorldPop projects [2]. For model inputs, population data are log-linearly interpolated to the three modelled time points (most recent household survey, current period, short-term projection) and adjusted such that aggregated district populations match the Spectrum population size by sex and five-year age group.

### Household survey

Household survey data inputs consist of data about HIV prevalence and, if available, ART coverage and biomarker-based recent HIV infection status from the most recently conducted national HIV household survey, for example a Demographic and Health Survey (DHS; https://dhsprogram.com/) or Population-based HIV Impact Assessment (PHIA; https://phia.icap.columbia.edu/) survey. Survey results are tabulated by district, sex, and five-year age group. If multiple household surveys were conducted nearly contemporaneously (e.g., both a DHS and PHIA in the same year), both may be used as data inputs, but surveys from earlier years, for example an earlier DHS round, are not included.

The smallest geographic stratification recorded in survey datasets is the survey strata, which usually correspond to first administrative divisions. When available, separate geographic datasets containing the latitude and longitude centroid of each survey cluster are used to assign respondent-level survey data to districts defined in the area hierarchy. Before releasing cluster coordinates, survey implementers geomask the locations to ensure respondent anonymity. Geomasking algorithms used for each survey are described in survey technical reports [3], and typically involve offsetting cluster centroids by up to ten kilometres depending on the urban/rural classification or population density.

Processing survey datasets to prepare model inputs involves assigning respondent-level survey data to districts based on the cluster geographic location, and tabulating survey-weighted proportions and the Kish effective sample size for each outcome (prevalence, ART coverage, recent infection) within district, sex, age group strata. Data on viral load suppression (VLS) among PLHIV may be used in place of ART coverage with a modelled adjustment for the assumed proportion of persons on ART who are virally suppressed.

### ANC testing and ART service delivery data

HIV service delivery data about HIV and ART among ANC clients and the total number currently receiving ART are extracted from national health management information systems (HMIS) or equivalent reporting systems. For ANC testing data, the following indicators are tabulated by district and calendar year: (1) the total number of ANC clients, (2) the number already known to be HIV positive prior to the first ANC visit for the current pregnancy (and not tested for HIV), (3) the number already on ART prior to first ANC visit, (4) the number tested for HIV at first ANC visit, and (5) the number tested HIV positive. It is important to ensure that each woman is only recorded once for each pregnancy. This is usually ensured by recording the HIV status at the first ANC visit separately from repeat HIV tests at follow-up ANC visits or repeat HIV tests. For ART service delivery data, input data are the total number receiving ART at health facilities in each district at the end of each calendar quarter, stratified by adult (15+ years) women, adult men, and children (0-14 years).

### Spectrum estimates

Results extracted from national or provincial Spectrum estimate files inform model parameters and constants for which district-level data are not available. These include patterns of HIV prevalence and ART coverage by age outside of the eligible age ranges for national household surveys, HIV survival and mortality probabilities, HIV incidence rate ratios by sex and age group, and children living with HIV arising from mother-to-child HIV transmission. Box 1 enumerates the indicators exported from Spectrum results.

## Process model

Primary model outcomes are HIV prevalence $\rho_{x,s,a,t}$, ART coverage $\alpha_{x,s,a,t}$, and the annual HIV incidence rate $\lambda_{x,s,a,t}$indexed by district $x$, sex $s\in\{F,M\}$, age $a\in\{0,5,10,\ldots,75,80\}$ representing five-year age groups 0-4, …, 80+, and time $t\in\{T1, T2, T3\}$. The time points represent the most recent national household survey with HIV testing (T1), the ‘current’ time period at which to generate estimates (T2), and a short-term projection typically nine months to one year ahead (T3), all referenced to the nearest calendar quarter (March, June, September, or December). Each district $x$ is located in a Spectrum file region $R_{x}$. For most countries which use a single national Spectrum file, the Spectrum region $R_{x}$ is the same for all districts. Some countries create national HIV estimates as an aggregation of multiple Spectrum files each representing first-level administrative regions (for example, provinces) [4]. In these cases, $R_{x}$ comprises the districts in the first administrative region represented by each subnational Spectrum file. The population size $N_{x,s,a,t}$ by district, sex, age group, and time is a constant fixed input.

### HIV prevalence and ART coverage at T1

Three linear models are specified for logit HIV prevalence, logit ART coverage, and log incidence rate at T1 (the time of the most recent household survey). HIV prevalence is modelled by

$$\mathrm{logit}\left( \rho_{x,s,a,T1} \right)=\beta_{0}^{\rho}+\beta_{S}^{\rho,s=M}+u_{a}^{\rho}+u_{a}^{\rho,s=M}+u_{x}^{\rho}+u_{x}^{\rho,s=M}+u_{x}^{\rho,a<15}+\eta_{R_{x},s,a}^{\rho}$$

where $\beta_{0}^{\rho}$ is the intercept, $\beta^{\rho,s=M}$ is the difference in logit HIV prevalence for men compared to women, $u_{a}^{\rho}\sim AR1(\sigma_{A}^{\rho},\phi_{A}^{\rho})$ is a random effect for the age pattern of HIV prevalence for women, $u_{a}^{\rho,s=M}\sim AR1(\sigma_{AS}^{\rho},\phi_{AS}^{\rho})$ is the difference in HIV prevalence for men compared to women age $a$, $u_{x}^{\rho}\sim BYM2(\sigma_{X}^{\rho},\phi_{X}^{\rho})$ is a spatial random effect capturing district-level variation in HIV prevalence among women, $u_{x}^{\rho,s=M}\sim BYM2(\sigma_{XS}^{\rho},\phi_{XS}^{\rho})$ captures district-level variation in difference between female and male prevalence, and $u_{x}^{\rho,a<15}\sim ICAR(0, \sigma_{XA}^{\rho})$ allows district-level variation in the ratio of paediatric prevalence to adult women prevalence. BYM2 refers to the re-parameterisation of the Besag-York-Mollie model proposed by Riebler et al. [5] with marginal standard deviation $\sigma>0$ and weight parameter $\phi\in(0, 1)$. ICAR refers to the intrinsic conditional autoregressive model of Besag and Kooperberg [6]. The offset $\eta_{R_{x},s,a}^{\rho}$ is a fixed input specifying assumed odds ratios for HIV prevalence outside the age ranges for which survey HIV prevalence data are available, typically above age 50 or age 65 and below age 15, depending on the survey. These are calculated based on Spectrum HIV prevalence estimates for region $R_{x}$.

Logit ART coverage is specified as

$$\mathrm{logit}\left( \alpha_{x,s,a,T1} \right)=\beta_{0}^{\alpha}+\beta_{S}^{\alpha,s=M}+u_{a}^{\alpha}+u_{a}^{\alpha,s=M}+u_{x}^{\alpha}+u_{x}^{\alpha,s=M}+u_{x}^{\alpha,a<15}+\eta_{R_{x},s,a}^{\alpha}$$

with terms defined analogously to the HIV prevalence model. If household survey data do not contain data on ART coverage by age, the offset $\eta_{R_{x},s,a}^{\alpha}$ is non-zero for all age groups and the age group random effect parameters $u_{a}^{\alpha}$ and $u_{a}^{\alpha,s=M}$ are not estimated due to non-identifiability. Identifiability constraints for specific data configurations are described below *Identifiability constraints*.

Model fixed effect terms ($\beta_{0}^{\rho}$, $\beta_{S}^{\rho,s=M}$, $\beta^{\alpha}$, $\beta_{S}^{\alpha,s=M}$) have diffuse $N(0, 5)$ prior distributions. Age patterns for prevalence and ART coverage ($u_{a}^{\rho}$, $u_{a}^{\rho,s=M}$, $u_{a}^{\alpha}$,$u_{a}^{\alpha,s=M}$) are modelled by first-order auto-regressive processes $AR1\left( \sigma,\phi\right)$ with marginal standard deviation $\sigma>0$ and correlation $\phi\in(-1,1)$. Standard deviation terms have a diffuse half-normal prior $N_{+}(0, 2.5)$, AR1 correlation parameters a $Unif\left( -1,1 \right)$ prior, and BYM2 weight parameters a $Beta\left( 0.5, 0.5 \right)$ prior.

### HIV incidence rate

The HIV incidence rate is modelled as a function of the HIV transmission rate, district-level HIV prevalence and ART coverage, and incidence rate ratios for incidence risk by sex and age group, reflecting basic HIV transmission dynamics that incidence risk depends on the probability of sexual contact with a partner with unsuppressed HIV infection in the local population. The log-linear model is a spatial analogue of the relation for modelling HIV incidence over time in the Estimation and Projection Package (EPP) model [7]:

$$\log\left( \lambda_{x,s,a,t} \right)=\beta_{0}^{\lambda}+\beta_{S}^{\lambda,s=M}+\log\left( \rho_{x,t}^{15-49} \right)+\log\left( 1-\omega\cdot\alpha_{x,t}^{15-49} \right)+u_{x}^{\lambda}+\eta_{R_{x},s,a,t}^{\lambda}$$

where $\beta_{0}^{\lambda}$ is proportional to the average HIV transmission rate for untreated HIV positive adults and $\beta_{S}^{\lambda,s=M}$ is the log incidence rate ratio for men compared to women. The terms $\rho_{x,t}^{15-49}$ and $\alpha_{x,t}^{15-49}$ are the HIV prevalence and ART coverage, respectively, among adults 15-49 years calculated by aggregating the sex/age-stratified quantities modelled above

$$\rho_{x,t}^{15-49}=\frac{\sum_{s\in\left\{ F,M \right\}} \sum_{a=15}^{45} N_{x,s,a,t}\cdot\rho_{x,s,at}}{\sum_{s\in\left\{ F,M \right\}} \sum_{a=15}^{45} N_{x,s,a,t}}$$

and

$$\alpha_{x,t}^{15-49}=\frac{\sum_{s\in\left\{ F,M \right\}} \sum_{a=15}^{45} N_{x,s,a,t}\cdot\rho_{x,s,at}\cdot\alpha_{x,s,at}}{\sum_{s\in\left\{ F,M \right\}} \sum_{a=15}^{45} N_{x,s,a,t}\cdot\rho_{x,s,at}}.$$

The term $\omega$ is the average reduction in HIV transmission rate per 1% increase in population ART coverage and is fixed at $\omega=0.7$ from the EPP model [7]. Random effects $u_{x}^{\lambda}\sim N(0,\sigma^{\lambda})$ with $\sigma^{\lambda}\sim N^{+}(0,1)$ allows data informed variation in district-level HIV transmission rate. The offsets $\eta_{R_{x},s,a,t}^{\lambda}$ specify log incidence rate ratios by sex and age group calculated from Spectrum model output, which are not estimable from current district-level input data.

If district-level data on recent HIV infection status from household surveys are not included in model estimation, the parameters $\beta_{0}^{\lambda}$, $\beta_{S}^{\lambda,s=M}$, and $u_{x}^{\lambda}$ are not estimated and the HIV incidence rate is a deterministic function of the HIV transmission rate and incidence rate ratios at time $t$ from Spectrum and the district level HIV prevalence and ART coverage. New HIV infections are not modelled below age 15 and mother-to-child HIV transmissions are not explicitly modelled, but the number of children living with HIV (CLHIV) are modelled based on the ratio of child to adult women prevalence and the number of children on ART.

### Short-term projection from T1 to T2 and T2 to T3

The HIV population is projected from the time of the most recent household survey (T1) to the current time (T2) based on the survival of PLHIV from Spectrum output, ageing from one five-year age group to the next, and the addition of new HIV infections calculated from the district-level HIV incidence rate and survival and ageing after infection.

Define the number of PLHIV at T1 as $H_{x,s,a,T1}=N_{x,s,a,T1}\cdot\rho_{x,s,a,T1}$. For age groups $a\geq5$, the number of PLHIV at T2 is modelled as

$$H_{x,s,a,T2}=H_{x,s,a,T1}\cdot S_{R_{x},s,a\to a,T1}+H_{x,s,a-5,T1}\cdot S_{R_{x},s,a-5\to a,T1}+\left( 1-e^{{-\Delta_{T1}\cdot\lambda}_{x,s,a,T1}} \right)\cdot\left( N_{x,s,a,T1}-H_{x,s,a,T1} \right)\cdot L_{R_{x},s,a\to a,T1}+\left( 1-e^{{-\Delta_{T1}\cdot\lambda}_{x,s,a-5,T1}} \right)\cdot\left( N_{x,s,a-5,T1}-H_{x,s,a-5,T1} \right)\cdot L_{R_{x},s,a-5\to a,T1}$$

where the terms correspond to:

- $S_{R_{x},s,a\to a,T1}$: the ratio for the number of PLHIV surviving from T1 to T2 and remaining in age group $a$ (that is did not age from $a$to $a+5$),
- $S_{R_{x},s,a-5\to a,T1}$: the survival ratio from T1 to T2 and ageing from age group $a-5$ to $a$,
- $\left( 1-e^{{-\Delta_{T1}\cdot\lambda}_{x,s,a,T1}} \right)$: the probability of acquiring HIV in between T1 and T2 period with duration $\Delta_{T1}$ for the susceptible population $N_{x,s,a,T1}-H_{x,s,a,T1}$,
- $L_{R_{x},s,a\to a,T1}$: the ratio surviving and remaining in age group $a$for those infected between T1 and T2,
- $L_{R_{x},s,a-5\to a,T1}$: the ratio surviving and aging from $a-5$to $a$ for those infected between T1 and T2.

The HIV survivorship ratios $S_{R_{x},s,a\to a',T1}$ for Spectrum region $R_{x}$are calculated from Spectrum results by the following steps:

1. Disaggregating single-year/single-age Spectrum model results for PLHIV at T1 and T2 and new infections between T1 and T2 to quarterly birth cohorts,
2. Subtracting the number of new infections within the cohort from the number of PLHIV at T2 to calculate the number of surviving PLHIV in each cohort.
3. Aggregate the survivors by quarter-age cohorts to those who survived and aged from $a$to $a'$ between T1 and T2, and calculate the ratio by dividing by the initial PLHIV $H_{x,s,a,T1}$.

The survivorship ratios are the same across all districts in Spectrum region $R_{x}$; variation in district ART coverage (or effectiveness) is assumed not to affect survival.

The survivorship ratios $L_{R_{x},s,a\to a',T1}$ for those newly infected are calculated by aggregating the number who were infected in each quarter-age cohort by the age group at infection $a$ and aged to $a'$ at T2 divided by incidence rate times susceptible population in age $a$ at T1.

For children below age $\Delta_{T1}$ at T2, the number of PLHIV are calculated based on the ratio $C_{R_{x},s,a,T1}$ of CLHIV to women PLHIV age 15–49 from Spectrum region $R_{x}$ applied to the district level PLHIV estimates:

$$H_{x,s,a,T2}=C_{R_{x},s,a,T1}\cdot\sum_{a^{'}=15}^{45} H_{x,F,a^{'},T1}$$

The ratio $C_{R_{x},s,a,T1}$ captures both mother-to-child transmission of HIV and subsequent survival of CLHIV between T1 and T2, but does not distinguish between these processes and implicitly assumes that these rates are the same for all districts $x\in R_{x}$, similar to the adult survivorship ratios.

The projection from T2 to T3 is calculated analogously to the projection from T1 to T2.

ART coverage at T2 is modelled through a linear model as the change in logit ART coverage between T1 and T2:

$$\mathrm{logit}\left( \alpha_{x,s,a,T2} \right)=logit\left( \alpha_{x,s,a,T1} \right)+\beta_{T_{2}}^{\alpha}+\beta_{T_{2}, s=M}^{\alpha}+u_{x,T2}^{\alpha}+u_{x,a<15,T_{2}}^{\alpha}+\eta_{R_{x},s,a,T2}^{\alpha}$$

where $\eta_{R_{x},s,a,T2}^{\alpha}$ is the offset for the logit change in ART coverage between T1 and T2 from Spectrum region $R_{x}$, $\beta_{T_{2}}^{\alpha}$ is the average change in logit ART coverage between T1 and T2 for adult women, $\beta_{T_{2},s=M}^{\alpha}$ is the difference in average change for men compared to women, $u_{x,T2}^{\alpha}\sim N(0,\sigma_{XT}^{\alpha})$ captures area-level variation in change in ART coverage, and $u_{x,a<15,T2}^{\alpha}\sim N(0, \sigma_{XA}^{\alpha})$ is difference in ART coverage change for children compared to adults.

ART coverage at T3 is modelled by applying the logit change in ART coverage between T2 and T3 from Spectrum region $R_{x}$ to each district $x\in R_{x}$:

$$\mathrm{logit}\left( \alpha_{x,s,a,T3} \right)=logit\left( \alpha_{x,s,a,T2} \right)+\eta_{R_{x},s,a,T3}^{\alpha}.$$

### ANC testing cascade

The number of ANC clients, HIV prevalence among pregnant women, and ART coverage among pregnant women by district and age are determined by (1) district-level HIV prevalence and ART coverage described above, and (2) the age-specific fertility rate (ASFR)and fertility rate ratios for HIV positive women and women on ART from Spectrum. For women aged 15-49 years ($a\in\{15, 20,\ldots, 45\}$), the predicted number of ANC clients $\Psi_{x,a,t}$ in district $x$, age $a$, and time $t$ is a log-linear model

$$\log\left( \Psi_{x,a,t} \right)=\log\left( N_{x,F,a,t} \right)+\psi_{R_{x},a,t}+\beta^{\psi}+u_{x}^{\psi}$$

with fixed inputs $N_{x,F,a,t}$ the female population size, $\psi_{R_{x},a,t}$ the ASFR in Spectrum region $R_{x}$ at time $t$. The parameter $\beta^{\psi}$ represents the log rate ratio for the number of ANC clients relative to the predicted fertility and random effects $u_{x}^{\psi}\sim N(0, \sigma^{\psi})$ capture district-level variation in the relative fertility. The parameters $\beta^{\psi}$ and $u_{x}^{\psi}$ capture multiple processes that give rise to differences between the predicted births and number of ANC clients, including district-level variation in ASFR, some pregnant women not attending ANC, foetal loss and still birth, or women attending ANC in different districts than their residence.

HIV prevalence $\rho_{x,a,t}^{ANC}$ and ART coverage $\alpha_{x,a,t}^{ANC}$ among pregnant women are modelled with analogous logit-linear models as the difference with district-level population prevalence $\rho_{x,F,a,t}$ and ART coverage $\alpha_{x,F,a,t}$, respectively:

$$\mathrm{logit}\left( \rho_{x,a,t}^{ANC} \right)= logit\left( \rho_{x,F,a,t} \right)+\beta^{\rho^{ANC}}+\beta_{t\in\{T2,T3\}}^{\rho^{ANC}}+u_{x}^{\rho^{ANC}}+u_{x, t\in\{T2,T3\}}^{\rho^{ANC}}+\eta_{R_{x},a,t}^{\rho^{ANC}}$$

$$\mathrm{logit}\left( \alpha_{x,a,t}^{ANC} \right)= logit\left( \alpha_{x,F,a,t} \right)+\beta^{\alpha^{ANC}}+\beta_{t\in\{T2,T3\}}^{\alpha^{ANC}}+u_{x}^{\alpha^{ANC}}+u_{x, t\in\{T2,T3\}}^{\alpha^{ANC}}+\eta_{R_{x},a,t}^{\alpha^{ANC}}.$$

The offsets $\eta_{R_{x},a,t}^{\rho^{ANC}}$ and $\eta_{R_{x},a,t}^{\alpha^{ANC}}$ are the log fertility rate ratio for HIV positive women compared to HIV negative women and for women on ART to HIV positive women not on ART, respectively, calculated from Spectrum model outputs for region $R_{x}$. The terms $\beta^{\theta^{ANC}}$represent the average difference between population and ANC outcomes at T1 (after removing the offset), and $\beta_{t\in\{T2,T3\}}^{\theta^{ANC}}$ represent the change in average difference from T1 to T2. Random effects $u_{x}^{\theta^{ANC}}\sim N\left( 0, \sigma_{X}^{\theta^{ANC}} \right)$ represent district-level variation in the relationship between general population and pregnant women outcomes and $u_{x t\in\{T2,T3\}}^{\theta^{ANC}}\sim N\left( 0, \sigma_{XT}^{\theta^{ANC}} \right)$ capture change between T1 and T2 in the district-level random effects.

### ART attendance

The number of PLHIV who are on ART by district $x$, sex $s$, age group $a$at time $t$are defined by the above model components as $A_{x,s,a,t}=N_{x,s,a,t}\cdot\rho_{x,s,a,t}\cdot\alpha_{x,s,at}$. To allow that some individuals may receive ART at facilities in a different district than they reside, we define the probability $\gamma_{x,x^{'},t}$ that a person on ART residing in district $x$ receives ART in district $x'$ at time $t$. It is assumed that individuals seek treatment in their district of residence, denoted $x'=x$, or a neighbouring district, denoted $x^{'}\sim x$. Thus, the probabilities $\gamma_{x,x^{'},t}$satisfy $\gamma_{x,x^{'},t}>0$ if $x^{'}\sim x$ or $x'=x$, $\gamma_{x,x^{'},t}=0$ if $x^{'}≁x$, and

$$\sum_{x^{'}\sim x,x^{'}=x} \gamma_{x,x^{'},t}=1.$$

The probabilities $\gamma_{x,x^{'},t}$ are parameterised as a multinomial logit model. For each district $x^{'}$, a parameter $\tilde{\gamma}_{x,t}$ is the log odds ratio of seeking ART the district $x'$ neighbouring $x$ compared to the district of residence $x$. Since the majority of ART clients typically seek treatment at facilities in their district of residence, the log odds ratios $\tilde{\gamma}_{x,t}$ are expected to be much less than zero, implying that the odds of seeking treatment in a neighbouring district is much less than one. The odds ratios will be larger in districts that ‘attract’ more ART clients from neighbouring districts and smaller in districts that do not attract clients. The parameters $\tilde{\gamma}_{x,t}$ are modelled as

$$\tilde{\gamma}_{x,t}=\tilde{\gamma}_{0}+u_{x}^{\tilde{\gamma}}+u_{x}^{\tilde{\gamma},t=T2}$$

$$\tilde{\gamma}_{0}=-4$$

$$u_{x}^{\tilde{\gamma}}\sim N\left( 0, \sigma_{X}^{\tilde{\gamma}} \right)$$

$$u_{x}^{\tilde{\gamma},t\in\{T2,T3\}}\sim N\left( 0, \sigma_{XT}^{\tilde{\gamma}} \right).$$

The intercept $\tilde{\gamma}_{0}=-4$ is the prior mean for the log odds of seeking ART in each neighbouring district compared to the home district. For a district with four neighbours, this implies a prior probability that 93% of ART clients obtain treatment in their home district. The random effects $u_{x}^{\tilde{\gamma}}$ is the district-level effect, and $u_{x}^{\tilde{\gamma},t\in\{T2,T3\}}$ is the change in the district-level log odds ratio between T1 and T2. Only a single value $\tilde{\gamma}_{x,t}$ is estimated for each district, which implies assumptions that (1) the odds of seeing treatment in district $x$ compared to the home district is the same for each neighbouring district, and (2) the probability of treatment seeking in a neighbouring district is the same for all sex and age groups.

The multinomial probabilities $\gamma_{x,x^{'},t}$ are calculated from the log odds ratios $\tilde{\gamma}_{x,t}$ via the softmax function

$$\gamma_{x,x^{'},t}=\frac{1}{1+\sum_{x^{*}\sim x} e^{\tilde{\gamma}_{x*,t}}}\left\{ \begin{aligned} 1 if x^{'}=x \\ e^{\tilde{\gamma}_{x*,t}} if x^{'}\sim x \\ 0 if x^{'}≁x \end{aligned} \right..$$

The number ART clients who reside in district $x$ and obtain ART in district $x'$ is

$$A_{x,x^{'},s,a,t}=A_{x,s,a,t}\cdot\gamma_{x,x^{'},t}=N_{x,s,a,t}\cdot\rho_{x,s,a,t}\cdot\alpha_{x,s,at}\cdot\gamma_{x,x^{'},t}.$$

The total number attending ART facilities in district $x^{'}$ by sex and age group, the quantity recorded in routine ART service delivery data, is

$$\tilde{A}_{x',s,a,t}=\sum_{x\sim x^{'}, x=x'} A_{x,x^{'},s,a,t}.$$

### Awareness of HIV status

The proportion of HIV positive adults aware of their HIV status $\nu_{x,s,a,t}$ for district $x$, sex $s$, age group $a$, and time $t$, is calculated as the sum of the district-level ART coverage $\alpha_{x,s,a,t}$ and the proportion of untreated adults $\tilde{\nu}_{R_{x},s,a,t}$ who are aware of their status, such that

$$\nu_{x,s,a,t}=\alpha_{x,s,a,t}+\frac{\tilde{\nu}_{R_{x},s,a,t}}{1-\alpha_{x,s,a,t}}.$$

Values for the proportion $\tilde{\nu}_{R_{x},s,a,t}$ of untreated adults who are aware of their status by sex, age group, and time are constant inputs taken from Shiny90 model results [8]. The Shiny90 model is applied as part of the Spectrum estimates process, and therefore these inputs are the same for all districts in the same Spectrum region $R_{x}$.

## Likelihood specification

Likelihood functions are specified for data sourced from national household surveys, routine ANC testing, and ART service delivery data. All outcomes are modelled at the most granular stratification of district, sex, and age group (see *Process model).* The data are frequently observed among coarser stratifications of each dimension. For notation, let {$x\}$ denote a collection of districts, $\left\{ s \right\}$ a collection of sexes, and $\left\{ a \right\}$ a collection of ages for which an outcome was observed. For example, ANC data are for pregnant women of childbearing age $\left\{ a \right\}=\{15, 20, 25, 30, 35, 40, 45\}$. ART data are sometimes recorded for both sexes together $\{s\} = \{F, M\}$ and for children $\left\{ a \right\}=\{0, 5, 10\}$ and adults $\left\{ a \right\}=\{15,\ldots, 80\}$. Survey data are occasionally available at the first administrative level comprising a collection of districts $\{x\}$.

### Household survey data

Household survey $v$ occurring at T1 furnishes weighted observations for HIV prevalence $\hat{\rho}_{\left\{ x \right\}\left\{ s \right\}\left\{ a \right\},v}$, ART coverage $\hat{\alpha}_{\left\{ x \right\}\left\{ s \right\}\left\{ a \right\},v}$, and the proportion recently infected $\hat{\kappa}_{\left\{ x \right\}\left\{ s \right\}\left\{ a \right\},v}$, with respective Kish effective sample sizes $M_{\left\{ x \right\}\left\{ s \right\}\left\{ a \right\},v}^{\hat{\rho}}$, $M_{\left\{ x \right\}\left\{ s \right\}\left\{ a \right\},v}^{\hat{\alpha}}$, and $M_{\left\{ x \right\}\left\{ s \right\}\left\{ a \right\},v}^{\hat{\kappa}}$. Multiplying these yield effect numbers of observed cases $Y_{\left\{ x \right\}\left\{ s \right\}\left\{ a \right\},v}^{\hat{\theta}}=M_{\left\{ x \right\}\left\{ s \right\}\left\{ a \right\},v}^{\hat{\theta}}\cdot\hat{\theta}_{\left\{ x \right\}\left\{ s \right\}\left\{ a \right\},v}$. The binomial distribution is used as a working likelihood for survey HIV prevalence and ART coverage observations:

$$Y_{\left\{ x \right\}\left\{ s \right\}\left\{ a \right\},v}^{\hat{\rho}}\sim Binomial(M_{\left\{ x \right\}\left\{ s \right\}\left\{ a \right\},v}^{\hat{\rho}}, \rho_{\left\{ x \right\}\left\{ s \right\}\left\{ a \right\},T1})$$

where

$$\rho_{\left\{ x \right\}\left\{ s \right\}\left\{ a \right\},t}=\frac{\sum_{x\in\left\{ x \right\}} \sum_{s\in\{s\}} \sum_{a\in\{a\}} N_{x,s,a,t}\cdot\rho_{x,s,a,t}}{\sum_{x\in\left\{ x \right\}} \sum_{s\in\{s\}} \sum_{a\in\{a\}} N_{x,s,a,t}}$$

and

$$Y_{\left\{ x \right\}\left\{ s \right\}\left\{ a \right\},v}^{\hat{\alpha}}\sim Binomial(M_{\left\{ x \right\}\left\{ s \right\}\left\{ a \right\},v}^{\hat{\alpha}}, \alpha_{\left\{ x \right\}\left\{ s \right\}\left\{ a \right\},T1})$$

where

$$\alpha_{\left\{ x \right\}\left\{ s \right\}\left\{ a \right\},t}=\frac{\sum_{x\in\left\{ x \right\}} \sum_{s\in\{s\}} \sum_{a\in\{a\}} N_{x,s,a,t}\cdot\rho_{x,s,a,t}\cdot\alpha_{x,s,a,t}}{\sum_{x\in\left\{ x \right\}} \sum_{s\in\{s\}} \sum_{a\in\{a\}} N_{x,s,a,t}\cdot\rho_{x,s,a,t}}.$$

The likelihood for the observed number recently infected depends on mean duration of recent infection (MDRI) $\Omega_{T}$ and false recent ratio (FRR) $\beta_{T}$ for the particular recent infection testing algorithm used in survey $v$. Based on the HIV incidence estimator from Kassanjee et al. [9], the predicted proportion recently infected among HIV positive persons is

$$\kappa_{x,s,a,t}=1.0-\exp\left( -\lambda_{x,s,a,t}\cdot\frac{1-\rho_{x,s,a,t}}{\rho_{x,s,a,t}}\cdot\left( \Omega_{T}-\beta_{T} \right)-\beta_{T} \right).$$

The working likelihood for the observed number recent $Y_{\left\{ x \right\}\left\{ s \right\}\left\{ a \right\},v}^{\hat{\kappa}}$ is

$$Y_{\left\{ x \right\}\left\{ s \right\}\left\{ a \right\},v}^{\hat{\kappa}}\sim Binomial\left( M_{\left\{ x \right\}\left\{ s \right\}\left\{ a \right\},v}^{\hat{\kappa}}, \kappa_{\left\{ x \right\}\left\{ s \right\}\left\{ a \right\},T1} \right).$$

Informative prior distributions based on known characteristics of the recent infection testing algorithm (RITA) are required for the MDRI and FRR parameters:

$$\Omega_{T}\sim N(\Omega_{T_{0}}, \sigma^{\Omega_{T}})$$

$$\beta_{T}\sim N\left( \beta_{T_{0}}, \sigma^{\beta_{T}} \right).$$

Prior parameters assumed for the analysis of recent Population-based HIV Impact Assessment surveys are $\Omega_{T_{0}}=130 \mathrm{days}$, $\sigma^{\Omega_{T}}=6.12 \mathrm{days}$, $\beta_{T_{0}}=0.0$, and $\sigma^{\beta_{T}}=0.0$.

### ANC testing data

Two years of ANC testing data are incorporated in model calibration: the year of the most recent survey $Y[T1]$ and the current year $Y[T2]$. ANC testing data observations are summarised as the total number of ANC clients $W_{\left\{ x \right\}Y[t]}^{ANC}$, the number of clients with HIV status ascertained $X_{\left\{ x \right\}Y[t]}^{ANC}$, the number HIV positive $Y_{\left\{ x \right\}Y[t]}^{ANC}$ (either known positive or tested positive), and the number already on ART prior to first ANC $Z_{\left\{ x \right\}Y[t]}^{ANC}$. In some cases, ANC testing data are only available for part of a given year, for example only the first three quarters January through September. Denote $M_{Y[t]}^{ANC}\in\{1,\ldots,12\}$ the number of months of reported data reflected in counts for year $Y[t]$.

The likelihood for the total number of ANC clients and ANC testing data is specified only for year $Y[T2]$ as a Poisson distribution with mean the sum of the model predicted clients within region $\left\{ x \right\}$ over the number of months reported:

$$W_{\left\{ x \right\}Y[T2]}^{ANC}\sim Poisson\left( \frac{M_{Y\left[ T2 \right]}^{ANC}}{12}\sum_{x\in\left\{ x \right\}} \sum_{a\in\left\{ 15,\ldots45 \right\}} \Psi_{x,a,T2} \right).$$

The observed number of HIV positive and already on ART among ANC clients at both $Y\left[ T2 \right]\left[ T1 \right]$ and $Y[T2]$ are modelled by a binomial distribution:

$$Y_{\left\{ x \right\}Y[t]}^{ANC}\sim Binomial\left( X_{\left\{ x \right\}Y\left[ t \right]}^{ANC},\rho_{\left\{ x \right\},\{15,\ldots49\},t}^{ANC} \right)$$

$$Z_{\left\{ x \right\}Y[t]}^{ANC}\sim Binomial\left( Y_{\left\{ x \right\}Y\left[ t \right]}^{ANC},\alpha_{\left\{ x \right\},\{15,\ldots49\},t}^{ANC} \right)$$

where the predicted prevalence and ART coverage are aggregated weighted by the predicted number of pregnant women by age $\Psi_{x,a,t}$:

$$\rho_{\left\{ x \right\}\left\{ a \right\},t}^{ANC}=\frac{\sum_{x\in\left\{ x \right\}} \sum_{a\in\{a\}} \Psi_{x,a,t}\cdot\rho_{x,a,t}^{ANC}}{\sum_{x\in\left\{ x \right\}} \sum_{a\in\{a\}} \Psi_{x,a,t}}$$

$$\alpha_{\left\{ x \right\}\left\{ a \right\},t}^{ANC}=\frac{\sum_{x\in\left\{ x \right\}} \sum_{a\in\{a\}} \Psi_{x,a,t}\cdot\rho_{x,a,t}^{ANC}\cdot\alpha_{x,a,t}^{ANC}}{\sum_{x\in\left\{ x \right\}} \sum_{a\in\{a\}} \Psi_{x,a,t}\cdot\rho_{x,a,t}^{ANC}}.$$

### Number receiving ART

ART service delivery data are reported as the number $\dot{A}_{\left\{ x \right\},\left\{ s \right\}\left\{ a \right\},t}$ receiving ART in the districts $\{x\}$, for sexes $\{s\}$, and ages $\{a\}$, at time $t\in\{T1,T2\}$. Where stratified data are available, ART input data are for both sexes ($\left\{ s \right\}=\{F,M\}$) for children ($\left\{ a \right\}=\{0,5,10\}$) and stratified by sex for adults ($\left\{ a \right\}=\{15,\ldots,80\}$). The observed number $\dot{A}_{\left\{ x \right\},\left\{ s \right\}\left\{ a \right\},t}$ is the sum of the number attending ART in district $x$ who reside in each neighbouring district $x^{'}$

$$\dot{A}_{\left\{ x \right\},\left\{ s \right\}\left\{ a \right\},t}=\sum_{s\in\{s\}} \sum_{a\in\{a\}} \sum_{x\in\{x\}} \sum_{x\sim x^{'}, x=x'} \dot{A}_{x^{'},x,s,a,t}.$$

Each of the unobserved counts $\dot{A}_{x^{'},x,s,a,t}$ follow a binomial distribution

$$\dot{A}_{x^{'},x,s,a,t}\sim Binomial\left( N_{x^{'},s,a,t}, \pi_{x^{'},x,s,a,t} \right).$$

where $\pi_{x^{'},x,s,a,t}=\rho_{x^{'},s,a,t}\cdot\alpha_{x^{'},s,a,t}\cdot\gamma_{x^{'},s,a,t}$.

The likelihood for the sum $\dot{A}_{\left\{ x \right\},\left\{ s \right\}\left\{ a \right\},t}$ is approximated using a normal distribution for the sum of binomial distribution

$$\dot{A}_{\left\{ x \right\},\left\{ s \right\}\left\{ a \right\},t}\sim N(\tilde{A}_{\left\{ x \right\},\left\{ s \right\}\left\{ a \right\},t}, \sigma_{\left\{ x \right\},\left\{ s \right\}\left\{ a \right\},t}^{\tilde{A}})$$

with

$$\tilde{A}_{\left\{ x \right\},\left\{ s \right\}\left\{ a \right\},t}=\sum_{s\in\{s\}} \sum_{a\in\{a\}} \sum_{x\in\{x\}} \sum_{x\sim x^{'}, x=x'} N_{x^{'},s,a,t}\cdot\pi_{x^{'},x,s,a,t}$$

$$\sigma_{\left\{ x \right\},\left\{ s \right\}\left\{ a \right\},t}^{\tilde{A}}=\sqrt{\sum_{s\in\{s\}} \sum_{a\in\{a\}} \sum_{x\in\{x\}} \sum_{x\sim x^{'}, x=x'} N_{x^{'},s,a,t}\cdot\pi_{x^{'},x,s,a,t}\cdot(1-\pi_{x^{'},x,s,a,t})}.$$

### Identifiability constraints

The identifiability of model terms depends on availability of data for a particular setting. In many applications, data are not available for all inputs. For example, many countries do not have household survey data about ART coverage or recent HIV infection, or in may countries district-level ART data are not available as far back as the most recent household survey.

In cases where some data inputs, some model parameters are fixed at default values and not estimated to ensure model identifiability:

- If survey data on ART coverage by age and sex are not available, the parameters $u_{a}^{\alpha}$ and $u_{a,s=M}^{\alpha}$ are fixed equal to zero and the average sex/age pattern of ART coverage is defined by the Spectrum offset $\eta_{R_{x},s,a}^{\alpha}$.
- If there are no ART data available at T1 (either survey or ART programme) or no ART data at T2, change in ART coverage over time is not identifiable from the data. The terms $\beta_{T_{2}}^{\alpha}$, $\beta_{T_{2}, s=M}^{\alpha}$, $u_{x,T2}^{\alpha}$, and $u_{x,a<15,T_{2}}^{\alpha}$ are fixed to zero and the equation for ART coverage at T2 is:

$$\mathrm{logit}\left( \alpha_{x,s,a,T2} \right)=logit\left( \alpha_{x,s,a,T1} \right)+\eta_{R_{x},s,a,T2}^{\alpha}.$$

- If no ART data (survey or ART programme) are available at T1 or T2, but data on ART coverage among ANC clients are available, the level of ART coverage is not identifiable, but ANC data informs spatial variation in ART coverage. In this case overall ART coverage is determined by the Spectrum offset, and only area random effects are estimated:

$$\mathrm{logit}\left( \alpha_{x,s,a,T1} \right)=u_{x}^{\alpha}+\eta_{R_{x},s,a}^{\alpha}$$

$$\mathrm{logit}\left( \alpha_{x,s,a,T2} \right)=logit\left( \alpha_{x,s,a,T1} \right)+u_{x,T2}^{\alpha}+\eta_{R_{x},s,a,T2}^{\alpha}$$

- If survey data on recent HIV infection are not included in model, the parameters $\beta_{0}^{\lambda}$, $\beta_{S}^{\lambda,s=M}$, and $u_{x}^{\lambda}$are fixed equal to zero. The sex ratio for HIV incidence is determined by the sex incidence rate ratio from Spectrum in the same years and the incidence rate in all districts is modelled assuming the same average HIV transmission rate for untreated adults, but varies according to district estimates for HIV prevalence and ART coverage.

## Case study data

Boundaries for the 28 districts and four metropolitan areas were constructed by aggregating Traditional Authority boundaries accessed from the Database of Global Administrative Areas (GADM) version 3.6 [10].

For household survey data, to produce publicly shareable demonstration data that reproduce case study results, we constructed approximate survey data inputs without reference to the geographic cluster location datasets for each survey. For the MDHS 2015-16, we relied on only data from the survey final report [11]. These consisted of HIV prevalence and corresponding sample size and standard error for adults aged 15-49 by the 28 districts and sex and the national-level HIV prevalence by five-year age group. Within each district, the number of HIV negative and HIV positive survey respondents were randomly distributed to five year age groups according to the distribution of HIV positive and HIV negative survey respondents at the national level.

MPHIA survey data were only geolocated to seven survey regions (five health zones with Lilongwe City and Blantyre City as separate survey region) [12]. To tabulate model inputs at the district level, we randomly allocated survey clusters in each region to district, proportional to district population size. This was similar to approaches used in other geospatial modelling exercises to approximately assign survey data to lower geographic levels when survey cluster geolocations were not available [13].

# Supplementary Figures

**
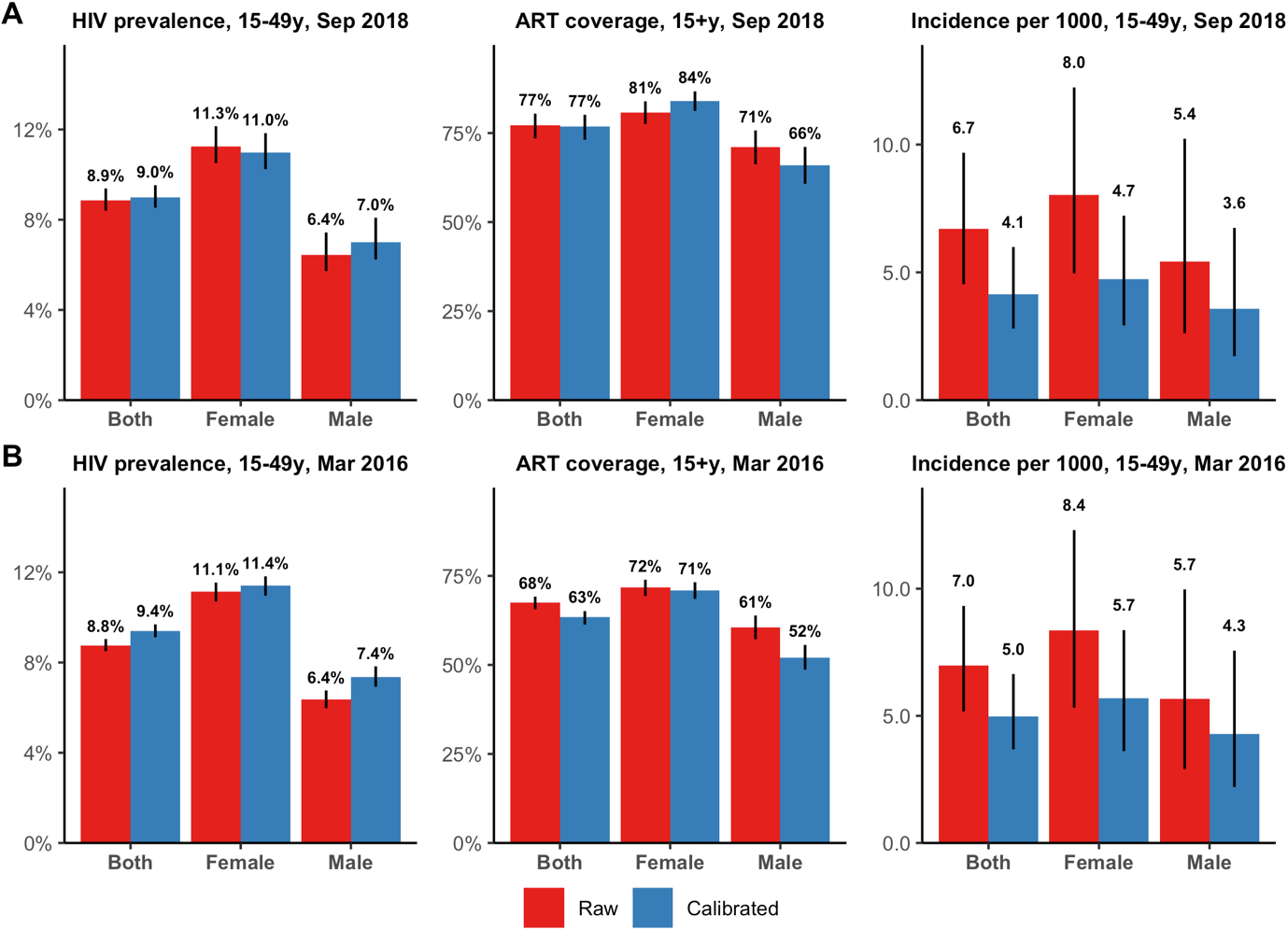
**

Figure S1. Comparison of raw uncalibrated Naomi results and results after calibration to Spectrum outputs by sex and coarse age groups (<15, 15+ years). Results represent national estimates for HIV prevalence among adults aged 15-49 years, ART coverage among aged 15 years and older, and HIV incidence rate per 1000 among aged 15-49 years for both sexes, males, and females in (A) September 2018, the current estimates period, and (B) March 2016, the baseline period corresponding to the most recent household surveys. ‘Raw’ results are the same as primary results shown in Figure 2 through Figure 5. Mean estimates for ‘calibrated’ results are the same as Spectrum estimates. Calibration was calculated using the logistic scaling method.

**
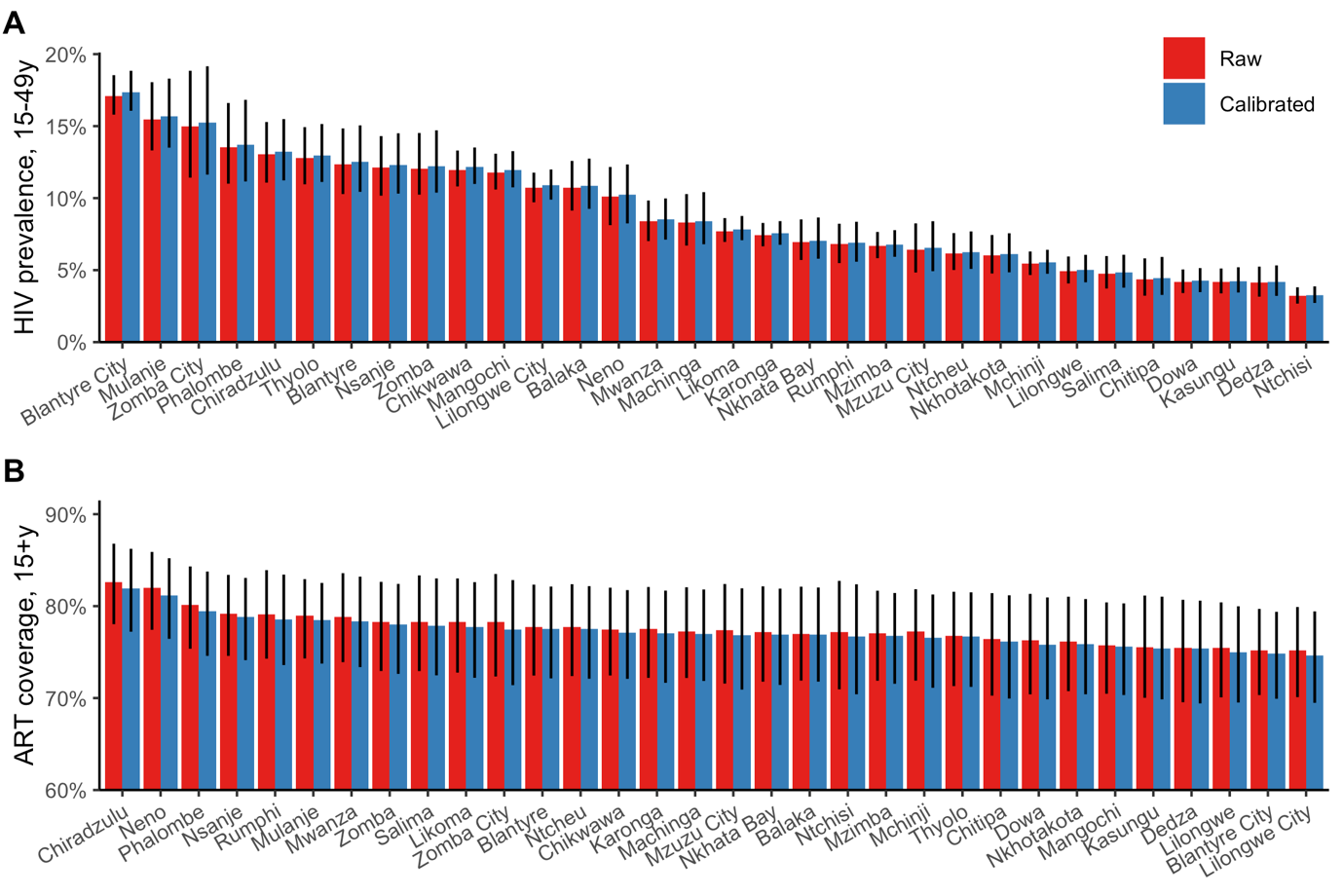
**

Figure S2. District-level comparison of raw uncalibrated Naomi results and results after calibration to Spectrum outputs by sex and coarse age groups (<15, 15+ years). Results represent September 2018. (A) District-level HIV prevalence for both sexes age 15-49 years. (B) District-level ART coverage for both sexes age 15+ years. ‘Raw’ results are the same as primary results shown in Figure 2 through Figure 5. Calibration was calculated using the logistic scaling method.

**
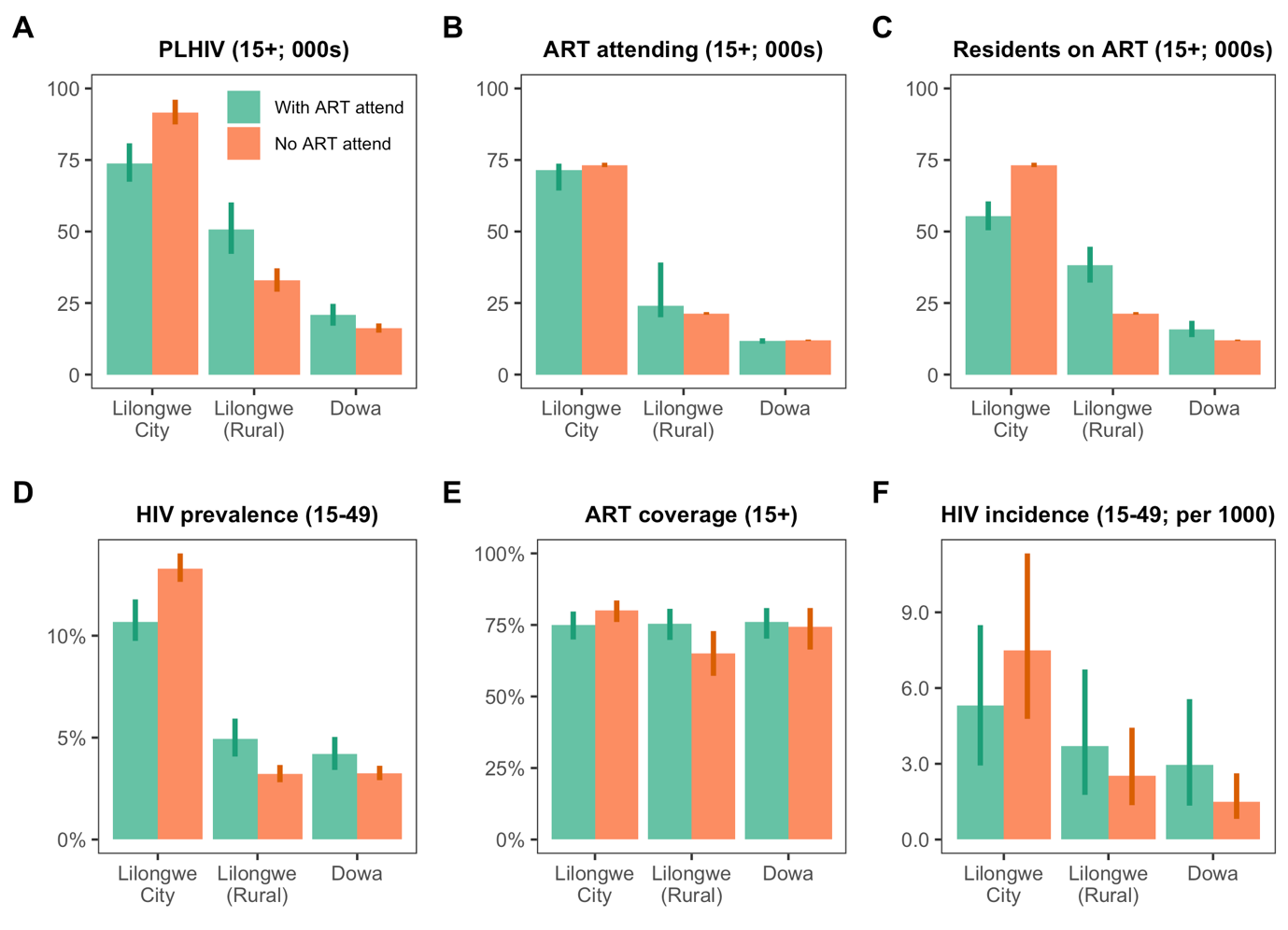
**

Figure S3. Comparison of full model results and results when assuming no probability of attending ART in neighbouring districts for Lilongwe City, Lilongwe District excluding the metropolitan area (Rural), and Dowa District. Results presented for September 2018. (A­–C) Vertical scale reflects the total number of adult (age 15 years and older) people living with HIV (PLHIV), number attending ART facilities in each district, and number of residents on ART in thousands. Results for HIV prevalence (D) and HIV incidence rate (F) are among adults aged 15–49 years and ART coverage (E) is among all adults 15 years and older. Bar heights represent posterior mean and vertical ranges indicate 95% credible intervals. Supplementary References

1. Center for International Earth Science - CIESIN - Columbia University. Gridded Population of the World, Version 4 (GPWv4): Population Count. In: NASA Socioeconomic Data and Applications Center (SEDAC) [Internet]. 2016 [cited 5 Mar 2021]. doi:10.7927/H4X63JVC

2. Tatem AJ, Garcia AJ, Snow RW, Noor AM, Gaughan AE, Gilbert M, et al. Millennium development health metrics: Where do Africa’s children and women of childbearing age live? Popul Health Metr. 2013;11. doi:10.1186/1478-7954-11-11

3. Burgert CR, Colston J, Roy T, Zachary B. Geographic Displacement Procedure and Georeferenced Data Release Policy for the Demographic and Health Surveys. In: DHS Spatail Analysis Reports No. 7 [Internet]. 2013 [cited 13 Mar 2021] p. 37. Available: https://dhsprogram.com/publications/publication-SAR7-Spatial-Analysis-Reports.cfm

4. Mahy M, Nzima M, Ogungbemi MK, Ogbang DA, Morka MC, Stover J. Redefining the HIV epidemic in Nigeria: from national to state level. AIDS. 2014;28 Suppl 4: S461-7. doi:10.1097/QAD.0000000000000456

5. Riebler A, Sørbye SH, Simpson D, Rue H, Lawson AB, Lee D, et al. An intuitive Bayesian spatial model for disease mapping that accounts for scaling. Statistical Methods in Medical Research. SAGE Publications Ltd; 2016. pp. 1145–1165. doi:10.1177/0962280216660421

6. Besag J, Kooperberg C. On Conditional and Intrinsic Autoregression. Biometrika. 1995;82: 733. doi:10.2307/2337341

7. Eaton JW, Brown T, Puckett R, Glaubius R, Mutai K, Bao L, et al. The Estimation and Projection Package Age-Sex Model and the r-hybrid model: new tools for estimating HIV incidence trends in sub-Saharan Africa. AIDS. 2019;33 Suppl 3: S235–S244. doi:10.1097/QAD.0000000000002437

8. Maheu-Giroux M, Marsh K, Doyle CM, Godin A, Lanièce Delaunay C, Johnson LF, et al. National HIV testing and diagnosis coverage in sub-Saharan Africa. AIDS. 2019;33: S255–S269. doi:10.1097/QAD.0000000000002386

9. Kassanjee R, McWalter TA, Bärnighausen T, Welte A. A new general biomarker-based incidence estimator. Epidemiology. 2012;23: 721–8. doi:10.1097/EDE.0b013e3182576c07

10. Database of Global Administratitve Areas (GADM). 2021 [cited 14 Feb 2021]. Available: https://biogeo.ucdavis.edu/data/gadm3.6/Rsf/gadm36_MWI_2_sf.rds

11. Malawi National Statistical Office (NSO), ICF. Malawi Demographic and Health Survey 2015-16. 2017 [cited 30 Apr 2019] p. 658. Available: https://dhsprogram.com/pubs/pdf/FR319/FR319.pdf

12. Ministry of Health Malawi, Centers for Disease Control and Prevention (CDC), University I at C. Malawi Population-based HIV Impact Assessment (MPHIA) 2015-16: Final Report. 2018.

13. Dwyer-Lindgren L, Cork MA, Sligar A, Steuben KM, Wilson KF, Provost NR, et al. Mapping HIV prevalence in sub-Saharan Africa between 2000 and 2017. Nature. 2019;570: 189–193. doi:10.1038/s41586-019-1200-9
